# General response of *Salmonella enterica* serovar Typhimurium to desiccation: A new role for the virulence factors *sopD* and *sseD* in survival

**DOI:** 10.1371/journal.pone.0187692

**Published:** 2017-11-08

**Authors:** Alice Maserati, Ryan C. Fink, Antonio Lourenco, Matthew L. Julius, Francisco Diez-Gonzalez

**Affiliations:** 1 Department of Food Science and Nutrition, University of Minnesota, Saint Paul, Minnesota, United States of America; 2 Department of Biology, Saint Cloud State University, Saint Cloud, Minnesota, United States of America; J Craig Venter Institute, UNITED STATES

## Abstract

*Salmonella* can survive for long periods under extreme desiccation conditions. This stress tolerance poses a risk for food safety, but relatively little is known about the molecular and cellular regulation of this adaptation mechanism. To determine the genetic components involved in *Salmonella*’s cellular response to desiccation, we performed a global transcriptomic analysis comparing *S*. *enterica* serovar Typhimurium cells equilibrated to low water activity (a_w_ 0.11) and cells equilibrated to high water activity (a_w_ 1.0). The analysis revealed that 719 genes were differentially regulated between the two conditions, of which 290 genes were up-regulated at a_w_ 0.11. Most of these genes were involved in metabolic pathways, transporter regulation, DNA replication/repair, transcription and translation, and, more importantly, virulence genes. Among these, we decided to focus on the role of *sopD* and *sseD*. Deletion mutants were created and their ability to survive desiccation and exposure to a_w_ 0.11 was compared to the wild-type strain and to an *E*. *coli* O157:H7 strain. The *sopD* and *sseD* mutants exhibited significant cell viability reductions of 2.5 and 1.3 Log (CFU/g), respectively, compared to the wild-type after desiccation for 4 days on glass beads. Additional viability differences of the mutants were observed after exposure to a_w_ 0.11 for 7 days. *E*. *coli* O157:H7 lost viability similarly to the mutants. Scanning electron microscopy showed that both mutants displayed a different morphology compared to the wild-type and differences in production of the extracellular matrix under the same conditions. These findings suggested that *sopD* and *sseD* are required for *Salmonella*’s survival during desiccation.

## Introduction

*Salmonella enterica*, a gram-negative bacterium belonging to the Enterobacteriaceae family, is a foodborne human pathogen that can cycle from the environment to animals and humans through their fecal matter [1–3]. Because of the wide variety of environments *Salmonella* can be exposed to, it can adapt to very diverse physical or chemical conditions. Generally, one of the most important factors impacting the ability of an organism to survive in a certain environment is the presence and, more importantly, the availability of water for chemical and biological reactions, a concept defined as water activity (a_w_). a_w_ is expressed as the ratio between the vapor pressure of water with a solute and the vapor pressure of pure water.

A relatively high a_w_ is essential for microbial growth, since at low a_w_ enzymatic reactions are inhibited and metabolism is reduced [4, 5]. Gram-negative bacteria such as *E*. *coli*, *Salmonella* and *Vibrio* require an a_w_ greater than 0.95 to grow [6]. As a result, one of the main strategies to control bacterial proliferation in food matrices is the reduction of a_w_ to create a low moisture environment [5, 7]. Most Enterobateriaceae are well adapted to persist in dry environmental conditions and *Salmonella* is no exception. To survive under harsh conditions, such as those found in a dry environment, bacteria need to activate a variety of cellular stress responses.

One of the first protection mechanisms activated by the shift from humid to dry environments is the response to osmotic stress induced by the decrease of water due to evaporation and the relative increase of the solute. In particular, this process makes the environment increasingly hypertonic, thus triggering specific molecular mechanisms that allow the cell to regulate the internal solute concentration to maintain the appropriate turgor [8–10]. Indeed, when exposed to low a_w_, *Salmonella* prevents and minimizes the loss of intracellular water and avoids membrane and protein damage due to the progressively hypertonic environment by increasing the influx of osmoprotectants. A recent study reported that up-regulation of osmoprotectant genes and operons such as *proP*, *proVWX*, and *osmU* can be observed after short-term desiccation on stainless steel coupons [11]. Up-regulation of some of these same genes was also observed after 2 h exposure to a_w_ 0.11 [12].

As aforementioned, osmotic protection is only one of the first mechanisms deployed by the cell in response to desiccation. A recent study from Gruzdev et *al*. suggested that it is likely to be part of a network of stress responses, such as oxidative and thermal stress, that act in a concerted fashion and modulate each other [13]. In fact, genes involved in the oxidative stress response through the formation and/or protection of iron-sulfur (Fe-S) clusters, such as *nifU*, *nifS*, *iscA*, part of the nitrogen fixation system (NIF), and *sufD* [14–18], have been found up-regulated after desiccation of *Salmonella* on Petri dishes [19]. In the same study, *fnr*, encoding for the fumarate and nitrate reduction protein and one of the master regulators for the metabolic shift from aerobic to anaerobic conditions, was also induced following desiccation. The knockout mutant Δ*fnr* showed impaired ability to survive dehydration and long-term storage at room temperature [19].

Interestingly, pre-exposure to desiccation has also been shown to induce protection against heat treatment [20–22]. Most of the theories concerning this cross-protection are based on physicochemical properties of the cell and focus on the stabilization of proteins during thermal exposure when less water is present [23–25]. However, some reports indicated that *Salmonella* thermal tolerance persists for a short period of time after rehydration [13]. Additionally, non-typhoidal *Salmonella* spp. and STEC *E*. *coli* showed higher tolerance to desiccation than non-pathogenic *E*. *coli* [23], although these species share almost identical physicochemical properties. These observations suggest that the thermal tolerance is not limited to chemical and physical phenomena, but could be in part a consequence of the complex network of overlapping stress responses induced by desiccation.

In addition to heat, exposure to desiccation has been linked with cross-protection for a multitude of other stressors in *Salmonella*, such as sodium hypochlorite, sodium chloride, bile salts, and hydrogen peroxide [13]. The ability of *Salmonella* to overcome different stresses is crucial for its virulence, since it is an essential capability during the infection process. To colonize the host, *Salmonella* has to survive extra- and intra-cellularly in the stomach and the intestine, where it is exposed to acid, osmotic, and oxidative stress, as well as starvation. Induction of virulence genes *hilA*, *invA*, and *spiC* was found after drying and storage in dry milk for short periods in *Salmonella* cells, both in planktonic and biofilm state [26].

The production of curli, thin aggregative fimbriae, cellulose, and lipopolysaccharides has also been reported to be important for survival under dry conditions [12, 27–29]. One of the hypothesis concerning the production of these cellular structures is that due to their high water retention quality, exopolysaccharides work as a water deposit [29]. Mutants in lipolysaccharides have been shown to be more sensitive to desiccation than parental *Salmonella* strains [29]. *Salmonella*’s ability to produce biofilm has also been reported to be important for desiccation survival on polypropylene discs [30].

In this work, we identify genetic components that are involved in the ability of *Salmonella*’s cells to survive reduction in moisture and exposure to very low water activity. In particular, two virulence genes, *sopD* and *sseD*, that are important for its survival to desiccation.

## Materials and methods

### Bacterial strains and culture preparation

The strains used in this study included *Salmonella enterica* serovar Typhimurium strain ATCC 14028 (*S*. *enterica* serovar Typhimurium), *Escherichia coli* O157:H7 strain ATCC 43895 (*E*. *coli* O157:H7), and two deletion mutants of the strain *S*. *enterica* serovar Typhimurium, Δ*sopD* and Δ*sseD*, obtained as described below by *camR* and *aphA-2* insertions, respectively. The stock cultures were stored at -55°C in a 5:1 solution of Luria-Bertani broth (LB; BBL, Detroit, MI) and glycerol. Working cultures were prepared in tryptic soy broth (TSB, Neogen, Inc., Lansing, MI) or 0.01 M glucose-supplemented LB broth (LBglc) from frozen stock cultures and grown overnight at 37°C shaking at 250 rpm. For mutant strain working cultures, chloramphenicol and kanamycin were added to LBglc to a final concentration of 50 and 100 μg/mL respectively.

### Inoculation on filters and RNA extraction

Working cultures of *S*. *enterica* serovar Typhimurium were freshly inoculated in TSB and grown for 3 h at 37°C shaking at 250 rpm. The cultures were collected through centrifugation (10 min at 4,696×g) and washed twice with distilled sterile water (DSW) to eliminate nutrient residues. Approximately 10^9^ CFU were spotted on 0.2 μm polycarbonate filters (Merck Millipore Ltd., Billerica, MA) and allowed to air-dry for 24 h at room temperature in a biosafety cabinet. Filters were placed in desiccators containing water or a saturated solution of lithium chloride 99% (Acros Organics, Thermo Fisher Scientific, Waltham, MA) to allow equilibration to a_w_ 1.0 and 0.11 respectively. Separate samples of corn starch were included as controls to monitor the a_w_ of the desiccators. After 4 days in the desiccators at 25°C, the a_w_ of corn starch samples were measured and the total RNA was extracted from *Salmonella* cells using the RNAprotect Bacteria Reagent and RNeasy Mini Kit (Qiagen, Hilden, Germany). The experiments were repeated three times in different days. Each time, three technical replicates were performed. The total RNA was extracted individually for each replicate and then the RNA was pooled together from all the replicates for the same conditions.

### RNA-Seq and global transcriptional analysis

Total RNA was processed at the University of Minnesota Genomic Center. Briefly, samples were quantified using a fluorimetric RiboGreen assay. Total RNA integrity was assessed using capillary electrophoresis with 2100 BioAnalyzer (Agilent Technologies, Santa Clara, CA), generating a RNA Integrity Number (RIN). Samples were converted to Illumina sequencing libraries using the TruSeq RNA Sample Preparation and Library preparation Kit (Illumina Inc., San Diego, CA). In summary, 1 μg of total RNA was reverse transcribed into cDNA. The cDNA was fragmented, blunt-ended, ligated to barcoded adaptors, and amplified using 15 cycles of PCR. Final library size distribution was validated using capillary electrophoresis and quantified using fluorimetry (PicoGreen) and via Q-PCR. Indexed TruSeq libraries were then normalized, pooled, hybridized to a paired end flow cell, and individual fragments were clonally amplified by bridge amplification on the Illumina cBot (Illumina Inc., San Diego, CA). Once clustering was completed, the flow cell was loaded on the HiSeq 2000 (Illumina Inc., San Diego, CA). Sequencing was performed on both strands.

Base call files for each cycle of sequencing were generated by Real-Time Analysis software (Illumina Inc., San Diego, CA). Primary analysis and de-multiplexing were performed using CASAVA software v1.8.2 (Illumina Inc., San Diego, CA). The end results of the CASAVA workflow, de-multiplexed FASTQ files, were analyzed using the DNASTAR SeqManNgen and ArrayStar softwares (DNASTAR, Inc., Madison, WI). The reads were assembled and mapped to the genome using SeqManNgen, while the levels of expression were estimated using ArrayStar.

The data discussed in this publication have been deposited in NCBI’s Gene Expression Omnibus [31] and are accessible through GEO Series accession number GSE86580 (http://www.ncbi.nlm.nih.gov/geo/query/acc.cgi?acc=GSE86580).

### Construction of deletion mutants

Two mutant strains were generated using the λ Red-mediated homologous recombination [32]. The plasmid pKD46 was introduced into *S*. *enterica* serovar Typhimurium using a MicroPulser electroporator (Bio-Rad Labs., Hercules, CA) at 1.7 kV. Primers used for knockout of *sopD* and *sseD* genes are reported in Table 1. All knockout primers were 60 nucleotides long, with 39 nucleotides homologous to the upstream or downstream regions flanking the targeted gene, and 21 nucleotides homologous to a universal cap designed in the drug resistance cassette (kindly provided by Dr. Roth, University of California at Davis, Davis, CA). The procedure used to make donor DNA fragments followed the protocol previously described [33]. Briefly, the PCR amplification protocol was: 95°C for 5 min; 95°C for 1 min, 55°C for 1 min, 72°C for 1:40 min × 30 cycles, and 72°C for 5 min. The 55°C melting temperature of our fragments was calculated on the 21-nucleotide caps.

**Table 1:** Primers used for λ Red-mediated recombination.

Gene	Primer ID	Sequence (5’-3’)^a^
*sopD*	*sopD*-F	CGGATATTGAATAATATAAATTTGAAGGAAAATATTATG**CACACAACCACACCACACCAC**
	*sopD*-R	TTATATTACTGACTATCTTTATGTCAGTAATATATTACG**CACCAAACACCCCCCAAAACC**
*sseD*	*sseD*-F	ATAGCTGGCTATCGCGCTTAATCTGAGGATAAAAATATG**CACACAACCACACCACACCAC**
	*sseD*-R	CTATTTCTTGCACCATGTTTACCTCGTTAATGCCCGGAG**CACCAAACACCCCCCAAAACC**

^a^Nucleotides homologous to the universal caps are in bold.

The *sopD* and *sseD* coding sequences were disrupted with the chloramphenicol (*camR*) and the kanamycin (*aphA-2*) resistance cassette, respectively, leaving the ATG region intact. The resistance cassettes were inserted in 3’-5’ to avoid polar effects of the universal cassette promoter on the downstream genes (S1 Fig).

The deletion of the *sopD* and *sseD* genes by substitution and insertion of the antibiotic cassette was verified by PCR amplification (S1 Fig) and Sanger sequencing. Primers used are listed in S1 Table. The sequenced reads were matched against NCBI database using the BLAST function [34] for the antibiotic cassette portion, while the gene’s upstream and downstream regions were identified using Artemis platform (Wellcome Trust Sanger Institute) [35]. The total genome of the two mutants was also extracted using the GenElute Bacterial Genomic DNA Kit (Sigma-Aldrich, St. Louis, MO) and sequenced on a HiSeq 2500 sequencer (Illumina, San Diego, CA). The assembling and mapping results were obtained using the DNASTAR SeqManNgen software (DNASTAR, Inc., Madison, WI).

### Growth curve

The growth rates and generation times were determined using the optical density (OD) measured at 600 nm with the Epoch 2 microplate reader (Bio Tek Instruments, Inc., Winooski, VT). Liquid cultures in LBglc were incubated overnight at 37°C with shaking at 250 rpm. The OD of each strain was then adjusted to 0.02 in LBglc and 200 μL aliquots were pipetted to 96-well plates. The plates were transferred to the plate reader with an incubation temperature of 37°C and orbital shaking. The ODs were recorded every 10 minutes for 24 h. Growth rates during the exponential phase were calculated by a regression of ln(OD) vs. time, where the slope was the growth rate based on the Monod equation (dN/dt = μN, where N is cell concentration expressed as OD, t is time, and μ is growth rate).

### Viability experiments on micro glass beads

Bacterial cultures were grown overnight at 37°C with shaking at 250 rpm in LBglc. The cultures were collected through centrifugation (10 min at 4,696×g) and washed twice with DSW to eliminate nutrient residues. For these experiments we decided to use glass beads (150–250 μm) (Corpuscular Inc., Cold Spring, NY). Our decision was motivated by the need to increase the number of cells, while still ensuring that the cells were evenly distributed and exposed to low a_w_. The greater total surface area offered by the beads compared to the filters allowed for a larger number of cells and for the formation of a thinner layer of adhering cells (as confirmed by the SEM micrographs). The washed pellets were re-suspended in 10 mL DSW and used to inoculate 10 g of sterile glass beads. Inoculated glass beads were spread on a sterile Petri dish and dried for 4 days at 38.5±0.5°C. For viable cell enumeration, 100 μL of each re-suspension were serially diluted in sterile saline (NaCl 0.9%), and 100 μL were spread plated on differential tryptic soy agar (dTSA) [TSA (Neogen, Inc.) supplemented with ammonium iron (III) citrate 16% (Fluka Analytical, Sigma-Aldrich, St. Louis, MO) (0.8 g/L) and sodium thiosulfate 98.5% (Acros Organics, Thermo Fisher Scientific, Waltham, MA) (6.8 g/L)].

After drying, the beads were distributed into sterile 200 μL PCR plastic tubes (Thermo Fisher Scientific). For exposure to a_w_ 0.11 and 1.0, the samples were equilibrated for 7 days at 25°C in desiccators containing a saturated lithium chloride (Acros Organics, Thermo Fisher Scientific) solution or sterile distilled water (SDW). After 7 days, the water activity of samples was measured (cutoff value: a_w_ reference ±0.02), and the samples were sealed. To determine the survival rate, beads for every sample were serially diluted in saline and spread plated on dTSA for cell enumeration. The recovery after every treatment was measured as cell viability in Log (CFU/g), and the survival rate was calculated as viability change in Log (CFU/g).

### Scanning electron microscopy

Samples were collected after inoculation on glass beads, dried for 4 days, and equilibrated 7 days to a_w_ 0.11 and 1.0. Immediately after collection, samples were fixed with a solution of 1% paraformaldehyde, 1% glutaraldehyde, and 0.05 M sodium cacodylate as previously described [36]. The samples were fixed overnight and then dehydrated through an ethanol series (10, 25, 70, 90, 95, and 100% ×2 for 24 h each) and HMDS series (30, 60 100%, 20 min each). Samples were transferred into 100% HDMS, air-dried for 48 h at room temperature, placed on 9.5 mm aluminum stubs with adhesive carbon tape, and coated with 20 nm of gold-palladium using the SC7620 Mini Sputter Coater (Quorum Technologies, Inc, Guelph, Canada). Scanning electron microscopy (SEM) imaging was performed with the JSM 6060LV scanning electron microscope (JEOL USA, Inc., Peabody, MA) using a 15 kV accelerating voltage.

### Statistics

Generation times for the wild-type and mutant strains were calculated averaging three independent biological replicates (*n* = 3). The generation time for each replicate was determined using the average OD of four technical replicates. All the viability experiments on glass beads were repeated at least four times (*n* ≥ 4) in different days (biological replicates). Each biological replicate consisted of three technical replicates. Technical replicate results were averaged to obtain the Log (CFU/g) for each biological replicate.

Significance, expressed as *p*-value, was calculated using a two-tailed Student’s t-test assuming equal variance for all experiments. Threshold for significance was set at *p* ≤ 0.05. Standard error of the mean (SE) was used to calculate the variation among samples. Averages, *p*-values, and SE were performed on the results of the biological replicates for each strain at each condition.

## Results

### Global transcriptional analysis

The transcriptional profile of *S*. *enterica* serovar Typhimurium cells air-dried and equilibrated to a_w_ 0.11 was compared to that of cells dried and equilibrated to a_w_ 1.0. Out of 4,489 genes [37] 719 (16%) were differentially expressed between the two conditions. Among these, 290 genes (40.3%) were up-regulated (2-fold cutoff) (S2 Table). We decided to focus on the up-regulated genes because are those most likely necessary for the adaptation to low water activity. These genes were categorized based on the KEGG Orthology (KO) database [38]. We found 5 functional classes: 1) metabolism (52 genes); 2) genetic information processing (24 genes); 3) environmental information processing (25 genes); 4) cellular processes (3 genes), and 5) infectious diseases (2 genes, both classified as virulence factors). The remaining 184 genes did not belong to any orthology group and were, therefore, unclassified. Table 2 lists a selected group of up-regulated genes and their functions by KO categories and sub-categories.

**Table 2:** Selected up-regulated genes in *Salmonella* exposed to a_w_ 0.11 versus 1.0.

	Locus	Gene name	Function	Fold change
**Ribosomal**				
	STM0095	*rluA*	23S rRNA/tRNA pseudouridine synthase A	4.45
	STM3441	*rpsJ*	30S ribosomal protein S10	3.42
	STM1835	*rrmA*	23S rRNA methyltransferase A	2.05
**Transporters**				
	STM0006	*yaaJ*	alanine/glycine transport protein	5.48
	STM3986	*trkH*	potassium transporter	3.70
	STM1491	*osmV*	proline/glycine betaine transport systems	2.91
	STM1379	*orf48*	amino acid permease	2.51
	STM2353	*hisQ*	histidine/lysine/arginine/ornithine transport protein	2.37
	STM1893	*znuB*	high-affinity zinc transporter membrane protein	2.28
	STM1806	*nhaB*	sodium/proton antiporter	2.28
	STM0835	STM0835	manganese transport transcriptional regulator MntR	2.05
	STM0568	*pheP*	phenylalanine transporter	2.05
**tRNAs**				
	STM3933	*leuT*	tRNA-Leucine	12.32
	STM3890	*gltU*	tRNA-Glutamate	10.50
	STM3932	*hisR*	tRNA-Histidine	5.48
	STM2394	*argW*	tRNA-Arginine	3.42
	STM4554	*leuP*	tRNA-Leucine	3.42
	STM0254	*aspU*	tRNA-Aspartate	3.19
	STM1134	*serX*	tRNA-Serine	3.08
	STM2824	STM2824	tRNA-Arg	2.05
	STM0674	*glnV*	tRNA-Gln	2.05
	STM4178	*gltV*	tRNA-Glu	2.05
	STM3037	*glyU*	tRNA-Gly	2.05
	STM2989	*metZ*	tRNA-Met	2.05
	STM4143	*tyrU*	tRNA-Tyr	2.05
**Transcription/translation regulators**				
	STM2836	*gutM*	DNA-binding transcriptional activator GutM	4.79
	STM1523	*yneJ*	transcriptional regulator	4.11
	STM0859	STM0859	transcriptional regulator	4.11
	STM1549	STM1549	translation initiation inhibitor	3.42
	STM2794	*ygaE*	DNA-binding transcriptional regulator CsiR	3.42
	STM4511	*yjiE*	DNA-binding transcriptional regulator	3.42
	STM3681	STM3681	transcriptional regulator	2.46
	STM1547	STM1547	transcriptional regulator	2.40
	STM1706	*yciH*	translation initiation factor Sui1	2.40
	STM3523	*glpR*	DNA-binding transcriptional repressor GlpR	2.33
	STM1773	*ychA*	transcriptional regulator	2.05
	STM3667	*yiaJ*	transcriptional repressor	2.05
	STM1488	*mlc*	pts operon transcriptional repressor	2.05
**DNA replication and repair**				
	STM0646	*holA*	DNA polymerase III subunit delta	4.11
	STM0263	*rnhA*	ribonuclease H	4.11
	STM0821	*dinG*	ATP-dependent DNA helicase DinG	3.73
	STM1821	*yoaA*	DNA helicase	3.42
	STM2496	*yfgE*	DNA replication initiation factor	3.42
	STM1201	*holB*	DNA polymerase III subunit delta'	2.74
	STM2223	*yejH*	ATP-dependent helicase	2.74
	STM1898	*ruvC*	Holliday junction resolvase	2.05
	STM0481	*priC*	primosomal replication protein N''	2.05
**Fimbriae**				
	STM4593	*sthB*	fimbrial usher protein	5.48
	STM1974	*fliK*	flagellar hook-length control protein	3.94
	STM0023	*bcfC*	fimbrial usher	3.42
	STM1913	*flhA*	flagellar biosynthesis protein FlhA	3.42
	STM1973	*fliJ*	flagellar biosynthesis chaperone	3.42
	STM4591	*sthE*	major fimbrial subunit	3.42
	STM0177	*stiA*	fimbrial subunit	2.05
	STM4594	*sthA*	fimbrial chaperone	2.05
**Virulence**				
	STM1399	*sscA*	secretion system chaperone	12.32
	STM1397	*sseA*	secretion system chaperone protein	3.70
	STM2945	*sopD*	secreted effector protein	3.13
	STM1170	*mviN*	virulence protein	3.03
	STM3764	*mgtC*	Mg2+ transport protein	2.40
	STM1401	*sseD*	translocation machinery component	2.05
**Membrane**				
	STM3178	*ygiY*	sensor protein QseC	3.42
	STM3372	*mreD*	rod shape-determining protein MreD	2.57
	STM3373	*mreC*	rod shape-determining protein MreC	2.22
	STM3374	*mreB*	rod shape-determining protein MreB	2.05

More specifically, multiple amino acid transporters where found up-regulated, including the alanine/glycine transporter *yaaJ*, the histidine/lysine/arginine/ornithine transporter *hisQ*, and the phenylalanine transporter *pheP*. Other genes involved with ion transportation were also found induced by low water activity: for example *trkH* for potassium, *znuB* for zinc, *nhaB* for sodium, and also the transcriptional regulator *mntR* for manganese. Several tRNA species were up-regulated: the tRNA for leucine (*leuP* and *leuT*), glutamate (*gltU*), histidine (*hisR*), arginine (*argW*), aspartate (*aspU*) and serine (*serX*) were the most abundant in low water activity. DNA replication genes, such as *holA*, *rnhA*, *dinG*, and DNA repair genes (*ruvC*, *priC*), were also induced in low water activity conditions.

Other up-regulated gene groups included transcriptional and translational regulators (i.e. *gutM*, *yneJ*, *yciH*) and ribosomal genes (i.e. *rluA*, *rpsJ*, *rrmA*). Genes involved in fimbriae and flagella biosynthesis were also up-regulated (i.e. *flhA*, *sthE*, *stiA*). Although we detected only two virulence genes by KO classification, *sopD* and *sseD*, four other known virulence factors were up-regulated in low water activity conditions. These virulence factors included *sscA*, a chaperon for the SseC translocon [39]; *sseA*, a class II chaperone specific for translocon proteins SseB and SseD [40–42]; *mviN*, involved in peptidoglycan biosynthesis and required for virulence in mice [43, 44]; and *mgtC*, part of the *mgtBC* operon in SPI-3, involved in the regulation of the membrane potential [45], and transcriptionally controlled by PhoP/PhoQ system [46].

### Δ*sopD* and Δ*sseD* mutant verification and sequencing

Based on the transcriptomic analysis results, we generated mutants in two virulence factors, *sopD* and *sseD*. Disruption of *sopD* by insertion of the chloramphenicol resistance cassette (*camR*) and of *sseD* by the kanamycin resistance cassette (*aphA-2*) was confirmed by PCR and sequencing. Genome-wide sequencing of the two mutants confirmed that the only variants present were localized in the targeted genes. When grown in LBglc at 37°C with aeration, both the mutants had the same generation time (G) compared to the wild-type (WT) strain: 22 min (SE 0.4 min) for WT, 21 min (SE 0.3 min) for Δ*sopD* (*p* = 0.33) and 21 min (SE 0.1 min) for Δ*sseD* (*p* = 0.11).

### Survival on glass beads

**Fig 1:**
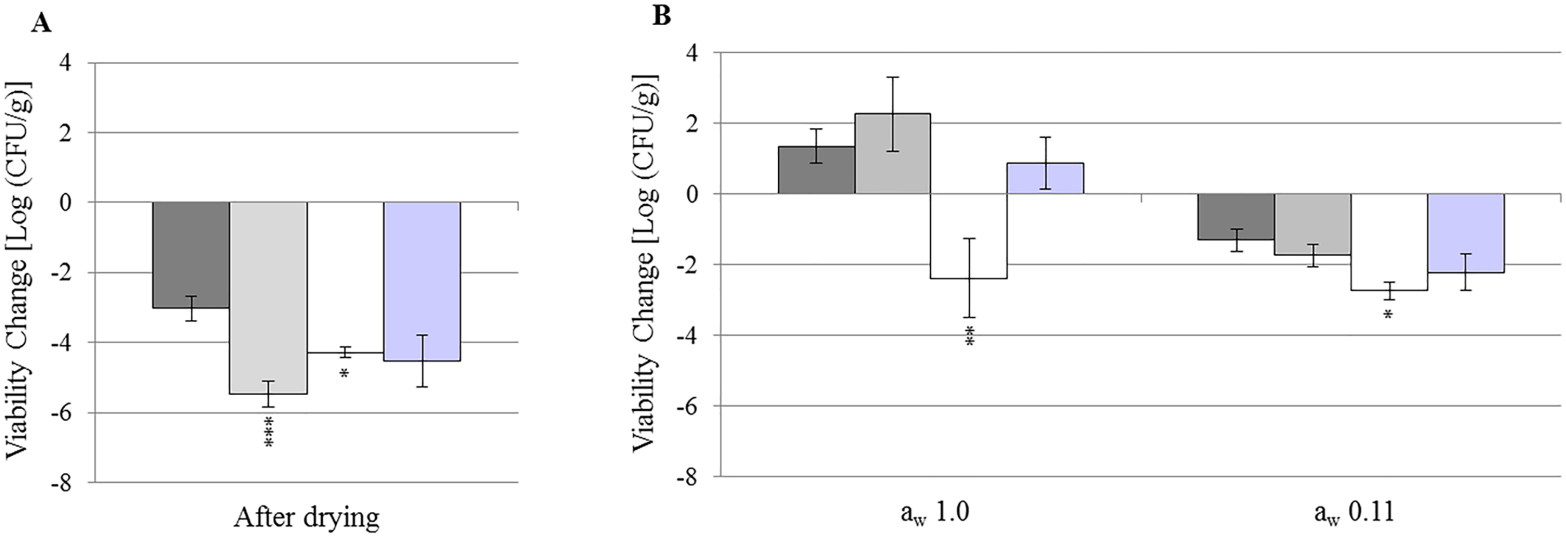
Changes in cell viability during drying and equilibration on glass beads. Changes in cell viability [Log (CFU/g)] of *S*. *enterica* serovar Typhimurium wild-type (WT; dark grey), Δ*sopD* (light grey), Δ*sseD* (white), and *E*. *coli* O157:H7 (purple) after 4 days drying (**A**) and after 7 days of equilibration to a_w_ 0.11 and 1.0 (**B**). Bars indicate standard error of the mean (SE). Stars indicate *p*-values < 0.05 (*), < 0.01 (**), and < 0.005 (***) in comparison with *Salmonella* WT under the same condition.

**Fig 2:**
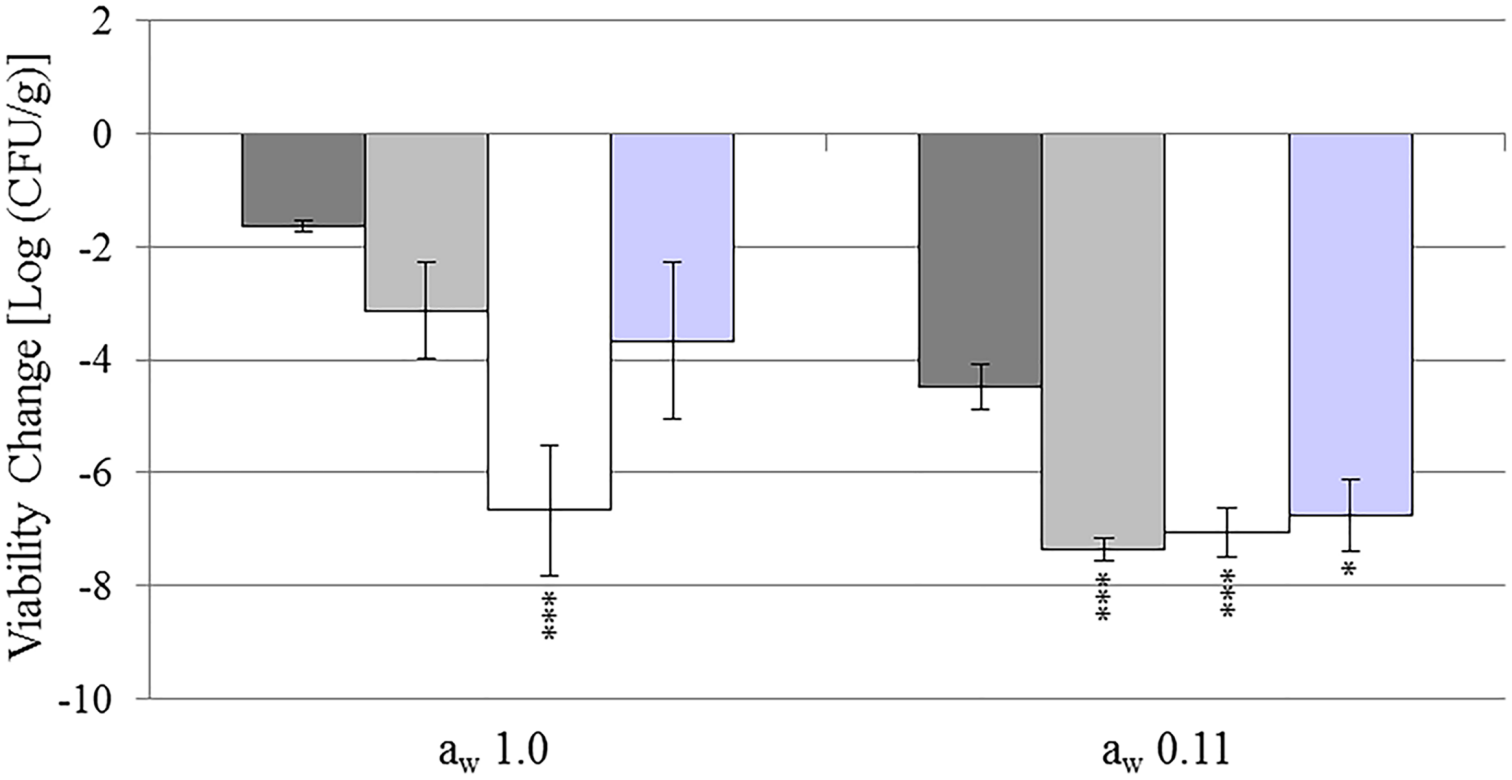
Total changes in cell viability during drying and equilibration on glass beads. Total changes in cell viability [Log (CFU/g)] of *S*. *enterica* serovar Typhimurium wild-type (WT, dark grey), Δ*sopD* (light grey), Δ*sseD* (white), and *E*. *coli* O157:H7 (purple) after 11 days of treatment (4 days drying and 7 days of equilibration to a_w_ 1.0 or 0.11). Bars indicate standard error of the mean (SE). Stars indicate *p*-values < 0.05 (*), < 0.01 (**) and < 0.005 (***) in comparison with *Salmonella* WT under the same condition.

To test the effect of *sopD* and *sseD* genes in response to desiccation, *S*. *enterica* serovar Typhimurium wild-type (WT), mutant strains, and *E*. *coli* O157:H7 were inoculated on micro glass beads, dried, and equilibrated to a_w_ 0.11 and 1.0 (Figs 1 and 2). As mentioned in the Materials and Methods, the use of glass beads was chosen to allow for a larger starting inoculum, while ensuring a homogenous surface distribution of the cells.

Cell counts decreased for all the strains after drying (Fig 1A) [*Salmonella* WT, -3.0 Log (CFU/g); Δ*sopD*, -5.5 Log (CFU/g); Δ*sseD*, -4.3 Log (CFU/g); and *E*. *coli* O157:H7, -4.5 Log (CFU/g)]. The differences between *Salmonella* WT and the mutants were significant (*p*-values 0.0003 for Δ*sopD* and 0.02 for Δ*sseD*). Although the *E*. *coli* O157:H7 count decreased, it was not significantly different from *Salmonella* WT (*p* = 0.06).

After equilibration to a_w_ 1.0 (Fig 1B), the cell counts for all the strains except Δ*sseD* increased compared to drying [*Salmonella* WT, 1.3 Log (CFU/g); Δ*sopD*, 2.3 Log (CFU/g); and *E*. *coli* O157:H7, 0.9 Log (CFU/g)]. Conversely, Δ*sseD* decreased by 2.4 Log (CFU/g). This difference was statistically significant when compared to the WT and Δ*sopD*, but not to *E*. *coli* O157:H7 (*p*-values 0.007, 0.014, and 0.06, respectively).

After equilibration to a_w_ 0.11 (Fig 1B), the cell counts for all the strains decreased compared to drying [*Salmonella* WT, -1.3 Log (CFU/g); Δ*sopD*, -1.7 Log (CFU/g); Δ*sseD*, -2.7 Log (CFU/g); and *E*. *coli* O157:H7, -2.2 Log (CFU/g)], although only Δ*sseD* had a significantly lower recovery than *Salmonella* WT (*p* = 0.012).

The large decrease in cell count of Δ*sopD* during drying lowered the cell count close to the detection limit of the experiment [2.7 Log (CFU/g)]. Therefore, it was difficult to estimate the additional decrease in viability and its significance after exposure to a_w_ 0.11. For this reason, we also calculated the total change in cell count for both a_w_ over the entire treatment period (11 days, Fig 2). At a_w_ 1.0, the total change in cell counts after the 11 day treatment was not significantly different between *Salmonella* WT, Δ*sopD*, and *E*. *coli* O157:H7 [*Salmonella* WT, -1.6 Log (CFU/g); Δ*sopD*, -3.1 Log (CFU/g); and *E*. *coli* O157:H7, -3.7 Log (CFU/g)]. On the contrary, Δ*sseD* had an overall reduction of 6.7 Log (CFU/g), significantly larger than *Salmonella* WT and Δ*sopD*, but not *E*. *coli* O157:H7 (*p*-values 0.0004, 0.03, and 0.14, respectively). At a_w_ 0.11 (Fig 2), we observed large total reductions in cell counts [*Salmonella* WT, -4.5 Log (CFU/g); Δ*sopD*, -7.4 Log (CFU/g); Δ*sseD*, -7.1 Log (CFU/g); and *E*. *coli* O157:H7, -6.8 Log (CFU/g)]. The mutants and *E*. *coli* O157:H7 had significantly lower cell viability compared to *Salmonella* WT (*p*-values 0.0001 for Δ*sopD*, 0.003 for Δ*sseD*, and 0.01 for *E*. *coli* O157:H7).

### Scanning electron microscopy of *S*. *enterica* serovar Typhimurium wild-type, Δ*sopD*, Δ*sseD*, and *E*. *coli* O157:H7

**Fig 3:**
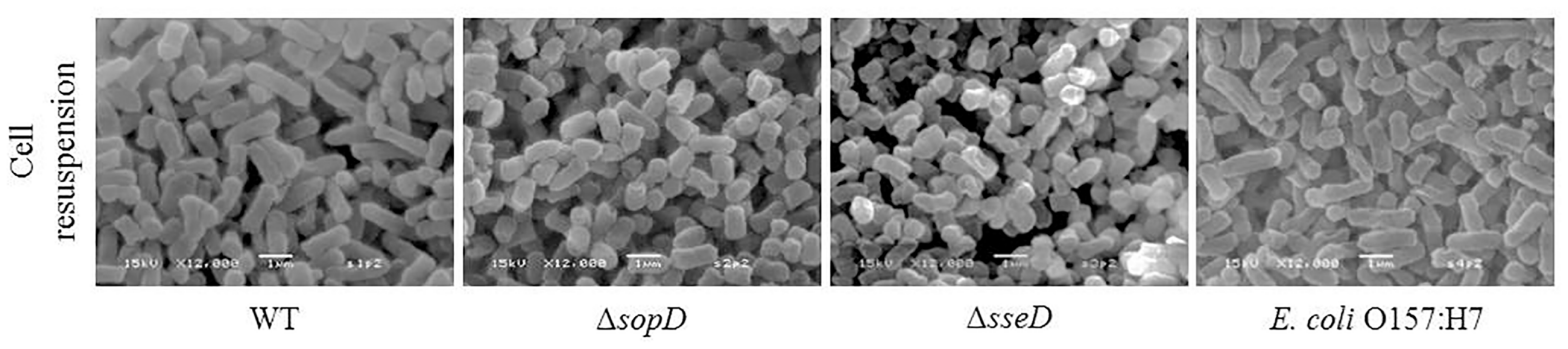
Scanning electron microscopy images of *S*. *enterica* serovar Typhimurium wild-type (WT), Δ*sopD* and Δ*sseD* strains, and *E*. *coli* O157:H7. Cells were collected from the overnight cell re-suspension used to inoculate the glass beads. The images show the change in cell morphology for the two mutants. Magnification and scale bar are embedded in the images.

Observations by SEM of *S*. *enterica* serovar Typhimurium wild-type (WT), Δ*sopD*, Δ*sseD*, and *E*. *coli* O157:H7 cells on glass beads showed WT and *E*. *coli* O157:H7 cells as rod-shaped cells with an average length of 1.5 μm and an average width of 0.5 μm. In contrast, both mutants displayed different cell morphology, more coccobacillary, with markedly smaller, rounder, and shorter cells, 1 μm in length or less (Fig 3).

**Fig 4:**
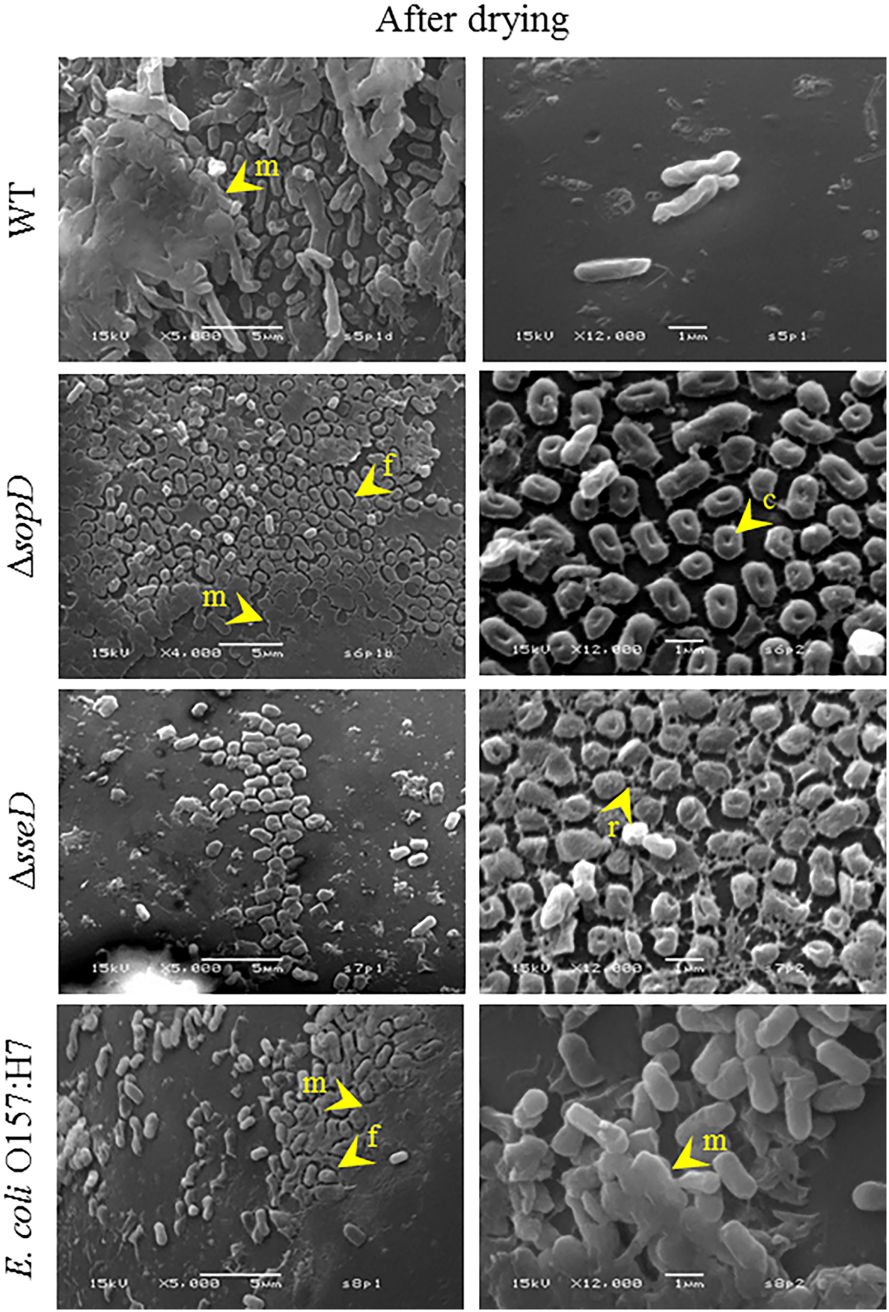
Scanning electron microscopy images of *S*. *enterica* serovar Typhimurium wild-type (WT), Δ*sopD* and Δ*sseD* strains, and *E*. *coli* O157:H7 after drying. Cells of *S*. *enterica* serovar Typhimurium WT, Δ*sopD*, Δ*sseD*, and *E*. *coli* O157:H7 inoculated and dried on glass beads for 4 days at 38.5°C. Note the differences in cell morphology and matrix structure among the four strains. Arrows and letters indicate specific elements present in each strain: matrix (m), fenestrations (f), cell concavity (c), and ropy filaments (r). The images are representative of the sample population. Magnification and scale bar are embedded in the images.

After drying, WT cells still appeared rod-shaped, although some cells displayed cell surface corrugations indicating a loss of turgidity (Fig 4). Cells were also embedded in a thick extracellular matrix. After drying, both mutants maintained a smaller size than the WT with a spheroidal shape and an evident indentation in the middle of the cell. Additionally, the cell density on the glass bead surface was lower than what was observed in the parental strain. Although Δ*sopD* also produced an extracellular matrix, it lacked the three dimensional structure observed for the WT. The matrix did not embed the cells but was attached to the bead surface. The matrix also had characteristic cell-shaped discontinuities where cells detached. In the same conditions, Δ*sseD* did not produce a homogeneous matrix, but presented an intricate network of ropy filaments. *E*. *coli* O157:H7 cells had morphology similar to *Salmonella* WT, but they produced a matrix similar to Δ*sopD*.

**Fig 5:**
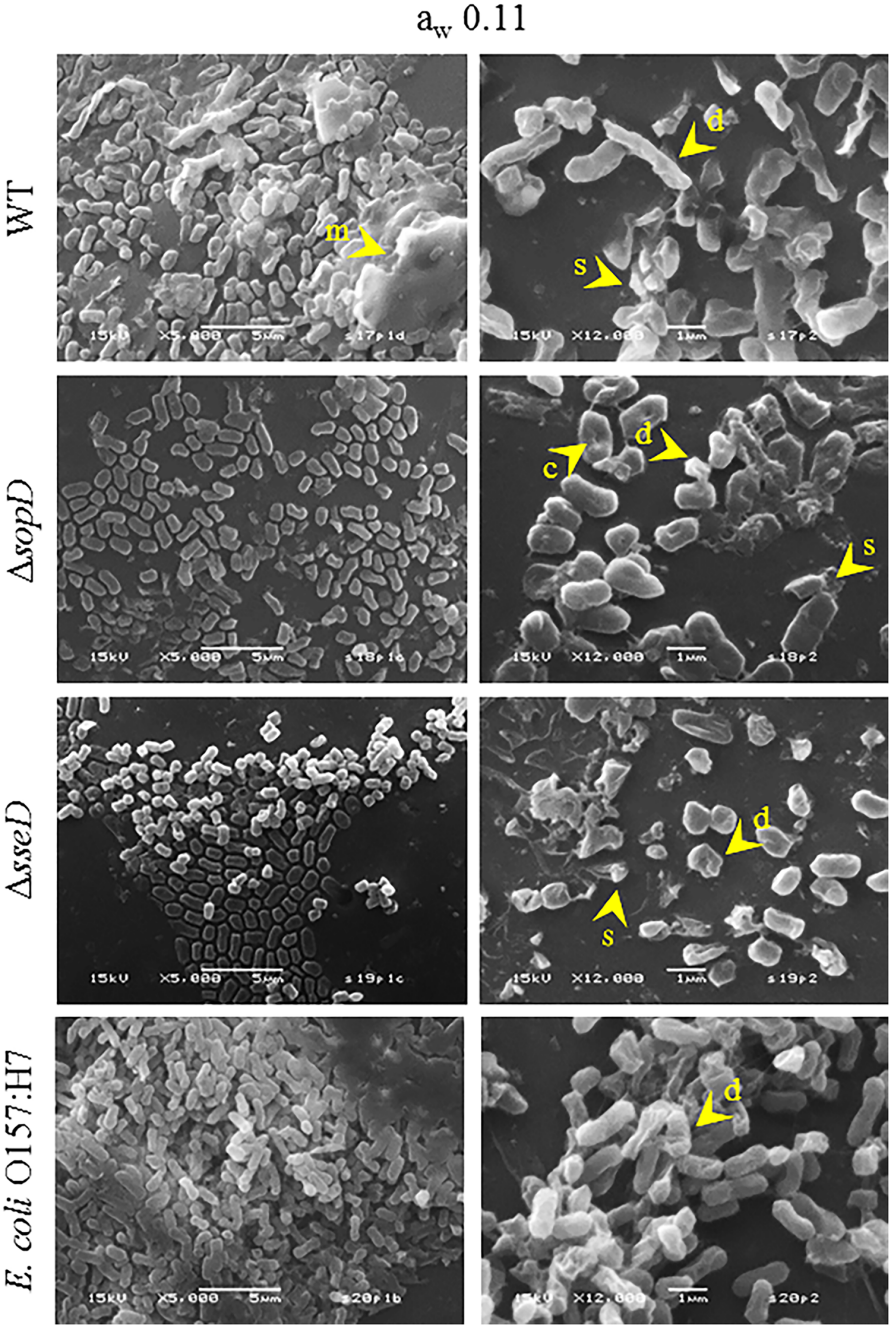
Scanning electron microscopy images of *S*. *enterica* serovar Typhimurium wild-type (WT), Δ*sopD* and Δ*sseD* strains, and *E*. *coli* O157:H7 after equilibration to a_w_ 0.11. Images of *S*. *enterica* serovar Typhimurium WT, Δ*sopD*, Δ*sseD*, and *E*. *coli* O157:H7 cells inoculated, dried and equilibrated for 7 days to a_w_ 0.11 on glass beads. Arrows and letters indicate specific elements present in each strain: matrix (m), cell concavity (c), damaged cells (d), and debris (s). The images are representative of the sample population. Magnification and scale bar are embedded in the images.

After 7 days of equilibration to a_w_ 0.11 (Fig 5), *Salmonella* WT cells were still characterized by the presence of an extracellular matrix, although the matrix was partially disrupted and detached. The cells maintained the rod morphology, but membrane corrugations and distortion, indicating loss of turgidity and cellular damage, were more evident than in the control. Both mutants, as well as *E*. *coli* O157:H7, lacked an extracellular matrix. Cells showed surface corrugation indicating membrane damage, and cell debris was present on the bead surface.

**Fig 6:**
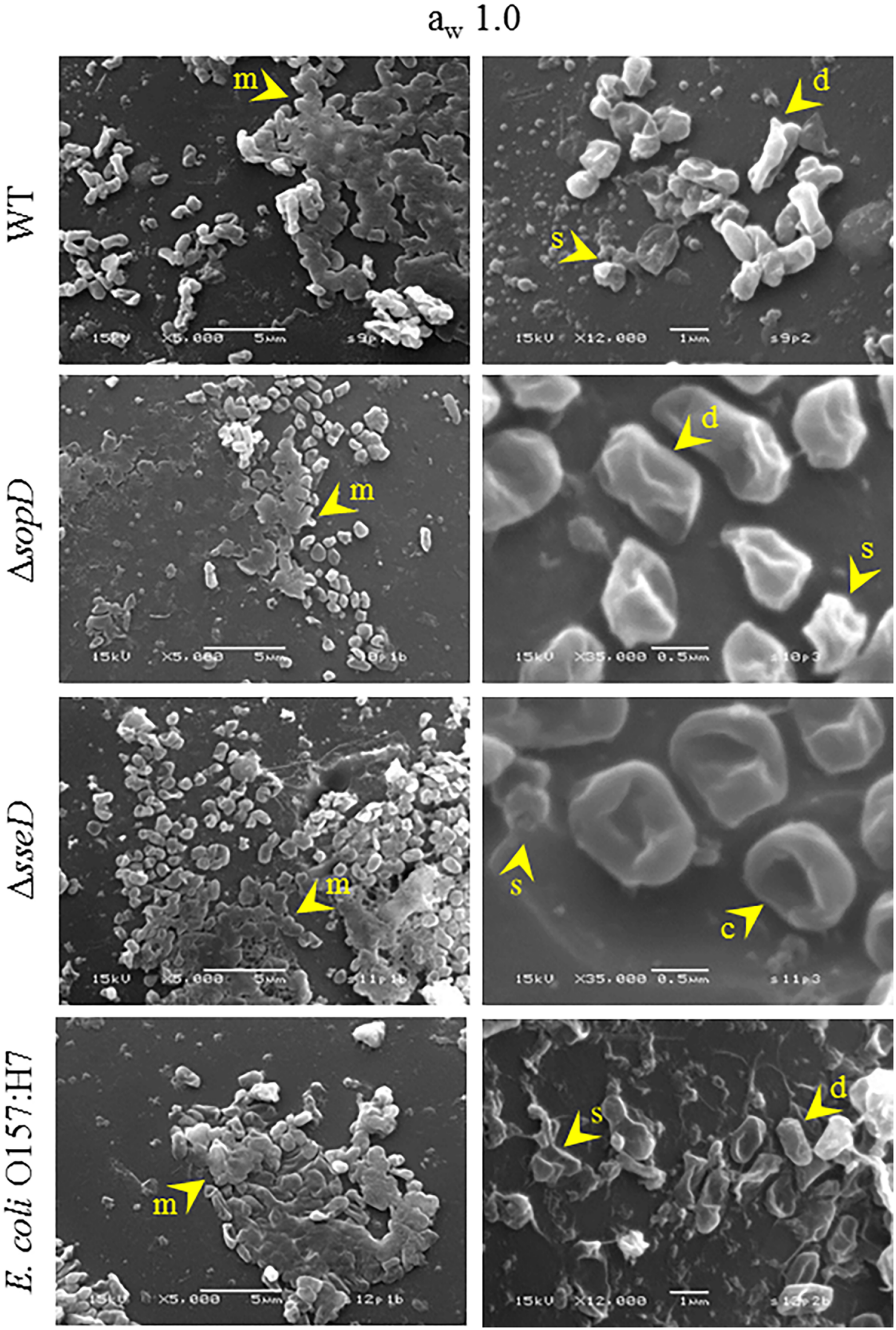
Scanning electron microscopy images of *S*. *enterica* serovar Typhimurium wild-type (WT), Δ*sopD* and Δ*sseD* strains, and *E*. *coli* O157:H7 after equilibration to a_w_ 1.0. Images of *S*. *enterica* serovar Typhimurium WT, Δ*sopD*, Δ*sseD*, and *E*. *coli* O157:H7 cells inoculated, dried and equilibrated for 7 days to a_w_ 1.0 on glass beads. Arrows and letters indicate specific elements present in each strain: matrix (m), cell concavity (c), damaged cells (d), and debris (s). The images are representative of the sample population. Magnification and scale bar are embedded in the images.

As observed at a_w_ 0.11, after equilibration for 7 days to a_w_ 1.0 (Fig 6) the two mutants maintained the characteristic morphology and presented loss of turgidity and wrinkling of the membrane, suggesting cell damage. Cell debris was also observed on the beads for both mutants as well as for the WT and *E*. *coli* O157:H7. Differently than at a_w_ 0.11, the two mutants had an extracellular matrix similar to the one formed by the WT and *E*. *coli* O157:H7.

## Discussion

The response of *S*. *enterica* serovar Typhimurium to desiccation and exposure to low a_w_ is extremely important because it can trigger other, seemingly unrelated, stress tolerance responses [13, 20, 21, 47, 48], but its underlying molecular mechanisms are still largely unknown. Several groups have performed different global transcription analysis on desiccated *Salmonella*. Deng et *al*. studied the transcriptome of this microorganism by RNA sequencing in low a_w_ peanut oil [49] and there are a good number of reports on microarray-based transcriptomic analyses on *Salmonella* survival or adaptation to abiotic surfaces such as filter paper, stainless steel, and plastic [11, 12, 19]. However, all of these studies have focused either on matrix-based low a_w_ or desiccation. To focus solely on the low a_w_ effect without the confounding factor of variations in the chemical composition of food matrices (e.g., batch to batch, sourcing of raw materials, and aging of the product), we decided to perform a global transcriptomic analysis on cells exposed to extreme low a_w_ using abiotic surfaces.

Our transcriptomic analysis showed that exposure to low a_w_ has a broad impact on the expression of many genes involved in anabolic and catabolic pathways. This was expected because the rate of enzymatic reactions slows when occurring in low a_w_, thus causing a decrease in the metabolic rate. This phenomenon has already been suggested in other studies [49]. However, we also found that low a_w_ induced the expression of many genes involved in DNA replication and repair. DNA damage is known to be an effect of desiccation, in particular covalent modifications and double-stranded breaks [50]. DNA repair genes were up-regulated under desiccation conditions in many different microorganisms, including *Deinococcus radiodurans* [51] and *Bradyrhizobium japonicum* [52]. We can speculate that during the shift to low a_w_, a portion of the replicating cells were unable to complete their replication due to either lack of energy and building blocks or water available for chemical reactions. This halt in the process would most likely be sensed as replication errors by the DNA replication checkpoints, thus inducing DNA repair mechanisms.

We also observed a stark increase in many tRNAs in cells exposed to low a_w_. tRNAs are usually difficult to observe by RNA sequencing, mainly because of their strong secondary and tertiary structures and post-transcriptional modifications [53]. Therefore, the observed increase in readable tRNA sequences for some tRNAs in desiccated cells might indicate that the post-transcriptional processing of those tRNAs is less efficient, possibly due to the same processes that cause DNA replication errors. However, an intriguing possibility is that the differential processing of tRNAs is a long-term adaptation strategy for the cell to coordinate amino acid transport and control translation. This would fit well with the high number of amino acid transporters and the transcriptional/translational regulators we found induced at low a_w_. Similar observations—up-regulation of amino acid transport and metabolism, and transcription and translation-associated genes—led Gruzdev et *al*. [19] to the conclusion that *de novo* protein synthesis is a requirement for the cell adapting to desiccation.

Osmolarity homeostasis is important in the desiccation process and, in our study, we observed the induction of *osmV* (2.91 fold), one of the genes involved in the transport of osmoprotectants during osmotic stress, in cells exposed to a_w_ 0.11 for four days. However, this was the only osmolarity-related gene induced in our treatment conditions. This is interesting, because one of the main long term responses the cell deploys to counteract osmotic stress during desiccation is the intracellular accumulation of osmoprotectants [54]. Osmoprotectants can be accumulated intracellularly from the medium or by *de novo* synthesis [55–57]. During desiccation, the ABC transporters ProU (ProVWX) and OsmU (OsmVWXY), in conjunction with the permease ProP, transport in the cytoplasm glycine/betaine and proline, the main osmoprotectants used by the cell [58–61]. Li et *al*. and Finn et *al*. found the ProU system up-regulated in *S*. *enterica* serovar Typhimurium desiccated on filter paper and stainless steel, respectively [11, 12].

The difference we observed in the induction of the ProU system may be explained by differences in methodology, including desiccation conditions and length of exposure to low a_w_. In particular, we washed and re-suspended the cells in pure water prior to desiccation, whereas Li et *al*. [12] used cells in PBS and Finn et *al*. [11] used cells in LB. As suggested elsewhere [19], the use of isotonic solutions in this kind of experiments can be problematic, as it would induce osmotic response during desiccation through the increase of solute concentration caused by water evaporation rather than through a decrease in a_w_. Moreover, those two studies focused on very short-term effects of desiccation (2 hours [12] and 4 hours [11]), whereas our analysis was performed after an extended period of time (4 days). Therefore, in our experiment, cells were already adapted to the initial osmotic shock and no longer needed many osmotic stress genes. These two non-exclusive explanations are corroborated by previous research that showed that 22 hour air-drying of water-resuspended *Salmonella* cells on plastic Petri dishes did not induce the Pro/Osm genes [19]. It is important to mention that production of the ProU transport system is usually induced by the presence of environmental glycine or betaine, and in our case, as well as in Gruzdev’s group, glycine was not present in the environment [62]. A similar explanation can be applied to the absence of up-regulation of the trehalose biosynthesis genes (*otsA* and *otsB*), which is another important osmoprotectant synthesized by the cell [63–65].

Our class of orthology gene classification of the differentially expressed genes identified two genes encoding for virulence factors, *sseD* and *sopD*. While *sseD* and *sopD* have been characterized for their role in virulence and infection mechanisms, to our knowledge, they have never been reported to be involved in desiccation adaptation and tolerance, and for this reason we targeted them for further analysis. SseD is a translocon component of the *Salmonella* Pathogenicity Island 2 (SPI-2) Type III Secretion System (T3SS) [66], and it is part of the *sseABCD* operon, encoding a chaperon protein and the other translocon components [67]. We also observed the induction of *sseA*, encoding the chaperonin for *sseB* and *sseD* [40–42], and *sscA*, encoding the chaperonin of *sseC* [39], in cells exposed to a_w_ 0.11. This indicates that the entire SPI-2 T3SS translocon is likely involved during adaptation and survival to desiccation and low a_w_. Interestingly, although SopD was initially identified as an effector translocated by the T3SS of SPI-1 [68], it is now suggested that the SPI-2 encoded injectisome can also be involved in its deployment [69]. Even though SopD release is activated under SPI-1 inducing conditions [70], *sopD* expression remains elevated during later stages of infection and is involved in survival and replication inside the macrophage [69, 71].

To better understand the impact of these two genes on the ability of *S*. *enterica* serovar Typhimurium to survive desiccation, we compared the survival rates and morphological characteristics of the WT, Δ*sopD* and Δ*sseD* mutants, and a virulent strain of *E*. *coli* (O157:H7). We chose to include *E*. *coli* in our comparisons because of its genetic and physiological relatedness to *Salmonella* and lack of the same kind of virulence mechanisms encoded by the SPIs.

The mutants were first compared to the WT under optimal growth conditions (LBglc, 37°C, shaking), and no differences were detected in generation time and in the ability to reach stationary phase. Additionally, the cell viability of the mutants in the initial cell-resuspension was checked, and the mutants, as well as *E*. *coli* O157:H7, had the same cell viability of *Salmonella* WT before being inoculated on glass beads (data not shown). On the contrary, interesting differences were detected when observing the different cell-resuspensions with SEM. Both mutants exhibited a shorter and coccoidal shape compared to the WT. Both Δ*sopD* and Δ*sseD* mutants had decreased tolerance to desiccation compared to the WT, clearly indicating that SopD and SseD play an important role during desiccation survival. To the best of our knowledge, this is the first time that different cell shape and size have been observed for mutants in these two genes.

In a previous study, field emission scanning microscopy (FESEM) was performed on a large number of SPI-2 effectors mutants and no changes in cell morphology were reported for Δ*sseD* in that case [72]. The use of a different *S*. *enterica* serovar Typhimurium strain—ATCC14028 instead of NCTC 12023—and different growth conditions might justify the different morphologies detected in our study. Furthermore, Chakravortty et *al*. used specific SPI-2 inducing conditions. As a result, the induction of other SPI-2 T3SS genes could have masked defects in cell morphology caused by the mutation in *sseD*.

A similar round shape was observed for *mreC* mutant in *Salmonella* [73] and *E*. *coli* [74]. This gene belongs to the *mreBCD* operon, which is responsible for the cell shape and correct formation of the cytoskeletons [75–79]. For *E*. *coli*, deletion in *mreBCD* caused the formation of spheroid-like cells defective in adjusting the rate of phospholipid synthesis [74]. Interestingly, we observed up-regulation of the entire *mreBCD* operon in WT cells of *S*. *enterica* serovar Typhimurium equilibrated to a_w_ 0.11, suggesting a role of the operon in the cell response to low a_w_, probably through the maintenance of a correct membrane composition and cell-shape. Although we cannot directly prove a connection between *sopD*/s*seD* and the *mre* operon, the fact that the two mutants, defective in cell-shape, are less tolerant to desiccation, suggests that the desiccation response mechanisms may include the *sopD*/*sseD*-dependent induction of the *mre* operon.

The effects of the mutations persisted after the additional week at a_w_ 0.11. The cell viability of the two mutants decreased once more. This indicates that *sopD* and *sseD* are not only involved in *Salmonella* adaptation to desiccation but are also essential for long period survival. It is interesting to note that drying and exposure to a_w_ 0.11 had a dramatic effect also on *E*. *coli* O157:H7, which lacks SPI-1 and 2 genes.

An additional possibility is that the mutants are more prone than the WT to enter a viable but not culturable state (VBNC). The VBNC state is described as a dormant state in which cells are metabolically inactive and are not culturable using standard methods [80]. This phenomenon has already been observed for *Salmonella* cells under different stress conditions, including drying and desiccation [81–83].

Structurally, after drying and prolonged exposure to a_w_ 0.11, *Salmonella* WT displayed a thick layer of solid extracellular matrix. In general, production of EPS has been associated to a higher desiccation tolerance in a variety of bacterial species, such as *E*. *coli*, *Acinetobacter calcoaceticus*, *Erwinia stewartii*, and *Rizhobium sullae* [84, 85]. It is believed that EPS works as a water reservoir and is protective from desiccation [86]. Moreover, genes encoding for fimbrial subunits were found induced in cells equilibrated to a_w_ 0.11 on filters—i.e. *sthA*, *sthB*, and *sthE*, part of the fimbrial operon *sthABCDE*, important for colonization in mice [87] as well as chickens [88]. The matrix observed for *Salmonella* WT after the 7-day equilibration showed some signs of damage, possibly due to the desiccation of the hydrogel.

After drying, the extracellular solid matrices of both *E*. *coli* O157:H7 and Δ*sopD* presented signs of damage as well as cell detachment, while after exposure to a_w_ 0.11, neither Δ*sopD* nor *E*. *coli* O157:H7 had any kind of extra-cellular material production. Interestingly, Δ*sseD* did not produce any solid matrix after drying, although cells were encased in a network of ropy filaments, which appeared to connect all cells. Δ*sseD* did not present these filaments after exposure to a_w_ 0.11 and the cell viability decreased compared to after drying.

It is possible that the mutant cells can temporarily produce extracellular structures with the role of protecting the cell from desiccation, but this response might be ineffective for long periods of exposure to low a_w_. Based on the different role and characteristics of SopD and SseD, we hypothesize that the decrease in desiccation and low a_w_ tolerance that we observed in our mutants is mainly due to lack of secretion of the effector protein SopD. As previously mentioned, SopD is an effector secreted by both T3SSs, while SseD is part of the injectosome of the SPI-2 T3SS. SPI-1 and SPI-2 T3SS are expressed during the infection process at different stages [89]. While SPI-1 expression is activated for the invasion of the host cell and the initial formation of the *Salmonella* containing vacuole (SCV), SPI-2 is necessary at a later stage, for *Salmonella* induced filaments (SIF) formation and bacterial replication inside the SCV.

In the case of the Δ*sopD* mutant, SopD cannot be produced nor secreted, and cell viability decreases dramatically from the first treatment (desiccation). Δ*sseD* is less susceptible than Δ*sopD* to the effect of initial desiccation, but after prolonged exposure to a_w_ 0.11, the effect of *sseD* depletion becomes as dramatic as *sopD*. These data suggest that during the initial desiccation, the SopD effector could still be secreted in the Δ*sseD* mutant, probably by SPI-1 T3SS, but during long-term low a_w_ exposure, the role of SPI-2 T3SS may become fundamental, and due to malfunctioning of the injectsome in Δ*sseD*, SopD may no longer be secreted. Additionally, the induction in the WT of *sseD*, as well as *sseA* and *sscA*, in cells equilibrated to a_w_ 0.11 strongly indicates that the correct assembly and functioning of the SPI-2 T3SS is required for survival at extreme low a_w_ conditions.

When cells were exposed to high a_w_ (a_w_ 1.0) after drying, cell viability for the WT, Δ*sopD*, and *E*. *coli* O157:H7 increased. This may be due to the ability of the cells to utilize proteins and nutrients released by the dead cells still on the beads, similarly to what hypothesized by Gruzdev et *al*. [19]. The presence of dead cells and cellular debris was observed at SEM after equilibration to a_w_ 1.0. Additionally, it has also been suggested that residues of extracellular polymeric substances (EPS) formed during desiccation can serve as a source of nutrients [90, 91]. The Δ*sseD* mutant, instead, did not increase in cell viability after equilibration to a_w_ 1.0. The differences in recovery from desiccation between the two mutants could be linked to the different role of the two effectors. Possibly, the lack of a completely developed needle structure in Δ*sseD* results in defective secretion of several other effectors required for the recovery process. At a_w_ 1.0 no differences were detected in EPS production, confirming that the differences in the exopolymeric matrix formation between the WT and the mutants/*E*. *coli* O157:H7 are due to differences mainly in response to desiccation.

Very recently, the regulatory system of a 97 nt small antisense RNA, STnc3140, encoded on the negative strand of *sopD* and positioned in its coding region (from position +726 to +822), has been partially characterized in *Salmonella* [92–95]. This sRNA was first named SLasRNA0334 and was identified in 2012 by Ramachandran et *al*. using a combination of differential RNA-seq and *in silico* analysis [94]. A subsequent study published in 2013 by Kröger et *al*. renamed this antisense RNA STnc3140 and showed that this RNA interacted with the RNA chaperone Hfq specifically during the transition from late exponential to early stationary phase [95]. In 2016, Colgan et *al*. thoroughly characterized the regulatory networks of 280 sRNAs in 5 different conditions (mid exponential phase, intermediate exponential phase, early stationary phase, late stationary phase, and SPI-2 inducing) and found that STnc3140 is positively regulated by RpoS and Hfq, but is repressed by HilD and Fur [92]. In particular, the authors of that study observed more than 3-fold decrease in STnc3140 expression in RpoS and Hfq mutants in late stationary phase and early stationary phase, respectively, and more than 3-fold increase in HilD and Fur mutants in early stationary phase. In a different study, Smirnov et *al*. observed a decreased expression of STnc3140 also in a ProQ mutant of *Salmonella* [93]. In our mutant, this small RNA was removed along with the SopD coding sequence.

The absence of STnc3140 could be at least partially responsible for the low a_w_-sensitive phenotype observed in the Δ*sopD*. However, this hypothesis is unlikely based on the fact that i) none of the regulators reported in the above mentioned studies are differentially expressed in our conditions; ii) the induction, under low a_w_ conditions, of *sopD* and few other genes related to secretion (e.g., *sseD*, *sseA*, and *sscA*) was described in the WT strain first and not limited to STnc3140; and iii) the phenotypes of Δ*sopD* and *ΔsseD*, although clearly distinct, they shared several similarities (e.g., coccoidal shape, sensitivity to desiccation and low a_w_, impacted extracellular matrix production at low a_w_). Regardless, as more information about small ncRNAs becomes available, it will be interesting to unravel the effects of the SopD effector from the ones of STnc3140, possibly by reintroducing into a Δ*sopD* background only STnc3140 and its regulatory unit and complementing the Δ*sopD* mutation with codon bias-modified copy of the gene not encoding STnc3140.

## Supporting information

S1 Fig Schematic representation of the mutants and of the genomic regions amplified for PCR verification. Schematic drawing of the *S*. *enterica* serovar Typhimurium wild-type *sopD* and *sseD* gene knock-out mutations with the chloramphenicol resistance cassette and the kanamycin resistance cassette, respectively (A). The sites of λ Red-mediated homologous recombination are indicated with crossing lines, while the primers used for the creation of the cassette are indicated with arrows. UL1 and UR1 are the universal caps part of the drug-cassette kit by Dr. Roth Laboratory (University of California at Davis, Davis, CA). Figure B shows the collocation of the primers used in the PCR reaction for the verification of Δ*sopD* and Δ*sseD* mutants of *S*. *enterica* serovar Typhimurium.
(TIF)

S1 Table Primers used for PCR verification and sequencing of the Δ*sopD* and Δ*sseD* mutants. (PDF)

S2 Table List of genes differentially expressed (more than 2-fold change) in *S*. *enterica* serovar Typhimurium wild-type equilibrated to a_w_ 0.11 on filters. (PDF)
