# Supplementary figures and images for: General response of Salmonella enterica serovar Typhimurium to desiccation: A new role for the virulence factors sopD and sseD in survival
Source: PLoS One. 2017 Nov 8;12(11):e0187692. doi: 10.1371/journal.pone.0187692 (PMC5678696; doi:10.1371/journal.pone.0187692)

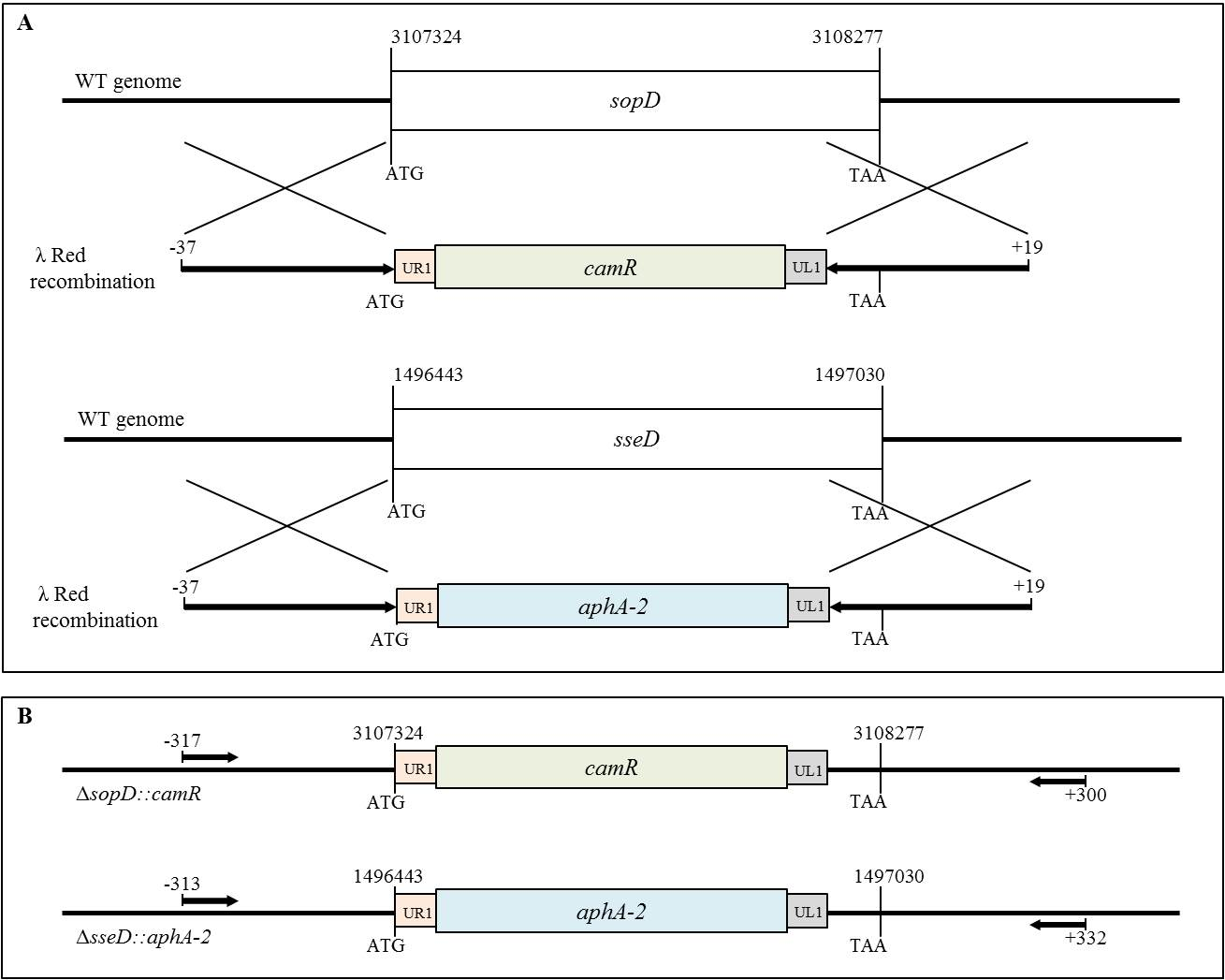

Supplement: S1 Fig — Schematic drawing of the S. enterica serovar Typhimurium wild-type sopD and sseD gene knock-out mutations with the chloramphenicol resistance cassette and the kanamycin resistance cassette, respectively (A). The sites of λ Red-mediated homologous recombination are indicated with crossing lines, while the primers used for the creation of the cassette are indicated with arrows. UL1 and UR1 are the universal caps part of the drug-cassette kit by Dr. Roth Laboratory (University of California at Davis, Davis, CA). Figure B shows the collocation of the primers used in the PCR reaction for the verification of ΔsopD and ΔsseD mutants of S. enterica serovar Typhimurium. (TIF) [file pone.0187692.s001.tif]
