# Supplementary material for: General response of Salmonella enterica serovar Typhimurium to desiccation: A new role for the virulence factors sopD and sseD in survival
Source: PLoS One. 2017 Nov 8;12(11):e0187692. doi: 10.1371/journal.pone.0187692 (PMC5678696; doi:10.1371/journal.pone.0187692)
Supplement: S1 Table — (PDF) [file pone.0187692.s002.pdf]

**Gene**	**Primer ID**	**Primer Sequence (5’-3’)**	**Direction**
sopD	*sopD*-350verifFW	CTTCAGAAATATTTACCCCACG	Forward
	sopD+350verifRV	GGCGTGTTTAAAGTGCTACC	Reverse
sseD	*sseD*-350verifFW	GAGGGATTGTTCATTTAAAGGC	Forward
	sseD+350verifRV	CAGGATGCGCAATAATTTCC	Reverse
