# Supplementary material for: General response of Salmonella enterica serovar Typhimurium to desiccation: A new role for the virulence factors sopD and sseD in survival
Source: PLoS One. 2017 Nov 8;12(11):e0187692. doi: 10.1371/journal.pone.0187692 (PMC5678696; doi:10.1371/journal.pone.0187692)
Supplement: S2 Table — (PDF) [file pone.0187692.s003.pdf]

Down-regulated genes			
**Locus**	**Gene name**	**Function**	**Fold change**
STM2970	*sdaC*	serine transport protein	-14.6
STM3975	*tatC*	twin-arginine protein translocation system subunit TatC	-13.1
STM0579	*ybdF*	hypothetical protein	-13.1
STM2256	*napB*	citrate reductase cytochrome c-type subunit	-13.1
STM1455	*ydgP*	electron transport complex protein RnfG	-12.4
STM4385	*ptxA*	PTS system L-ascorbate-specific transporter subunit IIA	-11.7
STM3473	*yhfC*	hypothetical protein	-11.7
STM1007	STM1007	hypothetical protein	-11.7
STM1045	STM1045	minor tail protein	-10.2
STM3665	*avtA*	valine--pyruvate transaminase	-10.2
STM2163	*yehX*	ABC-type proline/glycine betaine transport system ATPase component	-10.2
STM4557	*holD*	DNA polymerase III subunit psi	-10.2
STM0302	*safD*	fimbrial subunit	-8.8
STM0271	STM0271	hypothetical protein	-8.8
STM0813	*ybhP*	hypothetical protein	-8.8
STM0696	*ybfF*	hypothetical protein	-8.0
STM3054	*gcvH*	glycine cleavage system protein H	-8.0
STM0761	STM0761	fumarate hydratase	-7.3
STM4412	STM4412	permease	-7.3
STM1619	STM1619	cryptic aminoglycoside resistance gene	-7.3
STM1982	*rcsA*	colanic acid capsular biosynthesis activation protein A	-7.3
STM3930	*yifK*	transporter	-7.3
STM2799	*stpA*	DNA binding protein	-7.3
STM3830	*dgoR*	galactonate operon transcriptional repressor	-7.3
STM2832	*srlA*	glucitol/sorbitol-specific enzyme IIC component	-7.3
STM4019	*yihQ*	alpha-glucosidase	-7.3
STM3454	*slyX*	hypothetical protein	-6.8
STM2221	*bcr*	bicyclomycin/multidrug efflux system protein	-6.8
STM3378	STM3378	sulfite oxidase subunit YedZ	-6.8
STM3153	*yqhA*	hypothetical protein	-6.6
STM0361	STM0361	cytochrome BD2 subunit II	-6.3
STM2104	*cpsG*	phosphomannomutase	-6.3
STM1575	STM1575	transcriptional regulator	-6.3
STM0984	*msbA*	lipid transporter ATP-binding/permease	-6.3
STM1369	*sufA*	iron-sulfur cluster assembly scaffold protein	-6.1
STM2927	*surE*	stationary phase survival protein SurE	-5.8
STM3658	*yiaH*	inner membrane protein	-5.8
STM2106	*wcaI*	glycosyl transferase family protein	-5.8
STM3720	*yibR*	inner membrane protein	-5.8
STM2546	*suhB*	inositol monophosphatase	-5.8
STM0658	*ybeV*	molecular chaperone	-5.8
STM4104	STM4104	5'-nucleotidase	-5.8
STM0875	*rimK*	ribosomal protein S6 modification protein	-5.8

STM1675	STM1675	oxidoreductase	-5.8
STM1545	STM1545	multidrug efflux protein	-5.8
STM4566	*yjjI*	hypothetical protein	-5.8
STM1346	*ydiE*	hypothetical protein	-5.8
STM1247	STM1247	tRNA-Arg	-5.8
STM0435	*yajQ*	nucleotide-binding protein	-5.8
STM0432	*phnX*	phosphonoacetaldehyde hydrolase	-5.8
STM1471	*rstB*	sensor protein RstB	-5.8
STM1755	*ychJ*	hypothetical protein	-5.6
STM2023	*cbiM*	cobalt transport protein CbiM	-5.4
STM0206	*btuF*	vitamin B12-transporter protein BtuF	-5.1
STM3329	*yhcC*	FeS oxidoreductase	-5.1
STM1456	*rnfD*	electron transport complex protein RnfD	-5.1
STM3101	*yggT*	integral membrane protein	-5.1
STM0162	STM0162	inner membrane protein	-5.1
STM3960	*rhtB*	homoserine/homoserine lactone efflux protein	-5.1
STM0793	*bioA*	adenosylmethionine--8-amino-7-oxononanoate aminotransferase	-4.9
STM3959	*rhtC*	threonine efflux system	-4.9
STM1775	*hemK*	N5-glutamine S-adenosyl-L-methionine-dependent methyltransferase	-4.9
STM3016	*araE*	L-arabinose/proton symport protein	-4.7
STM1320	*ydjN*	kinase/transporter-like protein	-4.7
STM0502	*ybbL*	ABC transporter ATP-binding protein	-4.7
STM2816	STM2816	glycoporin	-4.4
STM1242	*envE*	envelope protein	-4.4
STM1975	*fliL*	flagellar basal body-associated protein FliL	-4.4
STM1418	*ssaQ*	type III secretion system protein	-4.4
STM2754	STM2754	hexulose 6 phosphate synthase	-4.4
STM3383	*prmA*	50S ribosomal protein L11 methyltransferase	-4.4
STM1343	*nlpC*	lipoprotein	-4.4
STM0948	STM0948	hypothetical protein	-4.4
STM1429	*ydhB*	DNA-binding transcriptional regulator	-4.4
STM2823	STM2823	tRNA-Arg	-4.4
STM2848	*hycF*	formate hydrogenlyase complex iron-sulfur subunit	-4.4
STM1223	*potC*	spermidine/putrescine ABC transporter membrane protein	-4.4
STM1154	*yceE*	drug efflux system protein MdtG	-4.4
STM2022	*cbiN*	cobalt transport protein CbiN	-4.4
STM1694	*sapC*	peptide transport protein	-4.4
STM4271	STM4271	inner membrane protein	-4.4
STM3160	STM3160	inner membrane protein	-4.4
STM1808	STM1808	hypothetical protein	-4.4
STM4139	*coaA*	pantothenate kinase	-4.4
STM3277	STM3277	inner membrane protein	-4.4
STM2937	*ygbF*	hypothetical protein	-4.4
STM4141	STM4141	hypothetical protein	-4.4
STM1856	STM1856	hypothetical protein	-4.4

STM1858	STM1858	hypothetical protein	-4.4
STM1037	STM1037	minor tail protein	-4.4
STM4276	STM4276	hypothetical protein	-4.4
STM1219	*ycfW*	outer membrane-specific lipoprotein transporter subunit LolE	-4.4
STM4401	*ytfG*	reductase	-4.4
STM0381	STM0381	inner membrane protein	-4.4
STM2308	*yfbB*	acyl-CoA thioester hydrolase YfbB	-4.4
STM2118	*wza*	outer membrane polysaccharide export protein	-4.4
STM0699	STM0699	hypothetical protein	-4.4
STM0138	*yacG*	zinc-binding protein	-4.4
STM2508	STM2508	hypothetical protein	-4.4
STM2527	STM2527	polyferredoxin	-4.4
STM0033	STM0033	5'-nucleotidase	-4.4
STM0087	*folA*	dihydrofolate reductase	-4.4
STM1725	*trpC*	bifunctional indole-3-glycerol phosphate synthase/phosphoribosylanthranilate isomerase	-4.1
STM2846	*hycH*	hydrogenase 3 large subunit processing protein	-4.1
STM1678	STM1678	2'-hydroxyisoflavone reductase	-4.1
STM2641	*nadB*	L-aspartate oxidase	-4.0
STM0758	*ybgR*	zinc transporter ZitB	-4.0
STM3120	STM3120	transcriptional regulator	-4.0
STM2691	STM2691	ABC transporter	-4.0
STM1123	STM1123	hypothetical protein	-4.0
STM0746	*tolR*	colicin uptake protein TolR	-4.0
STM2018	*cobU*	adenosylcobinamide kinase/adenosylcobinamide-phosphate guanylyltransferase	-3.9
STM0230	*rnhB*	ribonuclease HII	-3.9
STM1026	STM1026	hypothetical protein	-3.9
STM0806	*moaE*	molybdopterin guanine dinucleotide biosynthesis protein MoaE	-3.9
STM3825	*torT*	TMAO reductase system periplasmic protein TorT	-3.8
STM3725	*coaD*	phosphopantetheine adenylyltransferase	-3.8
STM3795	*ilvN*	acetolactate synthase 1 regulatory subunit	-3.8
STM2833	*srlE*	glucitol/sorbitol-specific enzyme IIB component	-3.8
STM2102	*wzxC*	colanic acid exporter	-3.7
STM1649	STM1649	hypothetical protein	-3.7
STM0547	*fimH*	minor fimbrial subunit	-3.7
STM0613	STM0613	hydrogenase protein	-3.7
STM0582	*ybdJ*	inner membrane protein	-3.7
STM1966	*yedF*	hypothetical protein	-3.7
STM0179	*yadE*	xylanase/chitin deacetylase	-3.7
STM0544	*fimI*	fimbrial protein	-3.7
STM3910	*ppiC*	peptidyl-prolyl cis-trans isomerase C	-3.7
STM0983	*ycaI*	hypothetical protein	-3.7
STM3976.S	*yigW*	DNase TatD	-3.7
STM1279	*yeaM*	regulatory protein	-3.7
STM1064	*pqiB*	paraquat-inducible protein B	-3.7

STM2673	*rplS*	50S ribosomal protein L19	-3.7
STM2900	*invH*	needle complex outer membrane lipoprotein precursor	-3.5
STM1133	STM1133	dehydrogenase	-3.5
STM1065	*ymbA*	outer membrane protein	-3.5
STM1983	*dsrB*	hypothetical protein	-3.5
STM3727	*rpmG*	50S ribosomal protein L33	-3.4
STM3529	*gldA*	glycerol dehydrogenase	-3.4
STM2034	*cbiB*	cobalamin biosynthesis protein	-3.4
STM0109	*yabN*	transcriptional regulator SgrR	-3.3
STM1993	*yedJ*	hypothetical protein	-3.3
STM2126	STM2126	multidrug efflux system subunit MdtA	-3.3
STM1817	*rnd*	ribonuclease D	-3.3
STM2803	STM2803	regulatory protein	-3.2
STM0589	*fepE*	ferric enterobactin transport protein FepE	-3.2
STM3620	*yhjQ*	cell division protein	-3.2
STM0165	*speD*	S-adenosylmethionine decarboxylase	-3.2
STM3316	*yrbI*	3-deoxy-D-manno-octulosonate 8-phosphate phosphatase	-3.2
STM3544	*yhhW*	hypothetical protein	-3.2
STM3848	*yidZ*	DNA-binding transcriptional regulator YidZ	-3.2
STM3364	*yhcP*	p-hydroxybenzoic acid efflux subunit AaeB	-3.2
STM2396	*pgtA*	activator	-3.1
STM2962	*gudT*	D-glucarate permease	-3.1
STM4284	*yjcO*	hypothetical protein	-3.1
STM3561	*livG*	leucine/isoleucine/valine transporter ATP-binding subunit	-3.1
STM2200	*lysP*	lysine transporter	-3.1
STM3692	*lldP*	L-lactate permease	-3.1
STM0292	STM0292	RHS-like protein	-3.1
STM0557	STM0557	inner membrane protein	-2.9
STM1698	STM1698	inner membrane protein	-2.9
STM1699	*ycjE*	hypothetical protein	-2.9
STM1843	STM1843	transporter	-2.9
STM2149	*stcD*	outer membrane lipoprotein	-2.9
STM0338	*stbC*	fimbrial usher	-2.9
STM3096	*yqgE*	hypothetical protein	-2.9
STM4113	*frwB*	PTS system fructose-like transporter subunit EIIB	-2.9
STM3066	*yggA*	arginine exporter protein	-2.9
STM0329	STM0329	isopropylmalate isomerase large subunit	-2.9
STM0084	STM0084	sulfatase	-2.9
STM2450	*amiA*	N-acetylmuramoyl-l-alanine amidase I	-2.9
STM1518	*marB*	hypothetical protein	-2.9
STM1745	*oppB*	oligopeptide transporter permease	-2.9
STM1526	*yneG*	hypothetical protein	-2.9
STM2617	STM2617	antiterminator-like protein	-2.9
STM3026	STM3026	outer membrane protein	-2.9
STM3024	*yohM*	nickel/cobalt efflux protein RcnA	-2.9

STM3143	*hybG*	hydrogenase 2 accessory protein HypG	-2.9
STM4551	STM4551	hypothetical protein	-2.9
STM1783.S	*pth*	peptidyl-tRNA hydrolase	-2.9
STM1579	*narW*	nitrate reductase 2 delta subunit	-2.9
STM0539	STM0539	inner membrane protein	-2.9
STM1156	*yceA*	hypothetical protein	-2.9
STM0523	*allB*	allantoinase	-2.9
STM3173	*plsC*	1-acyl-sn-glycerol-3-phosphate acyltransferase	-2.9
STM4082	*yiiQ*	hypothetical protein	-2.9
STM2863	*sitC*	permease	-2.9
STM0672	STM0672	inner membrane protein	-2.9
STM2535	*sseB*	enhanced serine sensitivity protein SseB	-2.9
STM2776	*iroE*	hydrolase	-2.9
STM1425	*ydhE*	multidrug efflux protein	-2.9
STM0884	*ulaA*	PTS system ascorbate-specific transporter subunit IIC	-2.9
STM4191	STM4191	hypothetical protein	-2.9
STM3823	*torC*	trimethylamine N-oxide reductase cytochrome c-like subunit	-2.9
STM0182	*panB*	3-methyl-2-oxobutanoate hydroxymethyltransferase	-2.9
STM0181	*panC*	pantoate--beta-alanine ligase	-2.9
STM0175	*stiC*	fimbrial usher	-2.9
STM2671	*yfiR*	hypothetical protein	-2.9
STM2263	*yojI*	multidrug transporter membrane protein/ATP-binding component	-2.9
STM1385	*ttrB*	tetrathionate reductase complex subunit B	-2.9
STM3942	STM3942	hypothetical protein	-2.9
STM3745	STM3745	hypothetical protein	-2.9
STM3780	*gatY*	fructose-1,6-bisphosphate aldolase	-2.9
STM4176	*purH*	bifunctional phosphoribosylaminoimidazolecarboxamide formyltransferase/IMP cyclohydrolase	-2.9
STM4589	*creC*	sensory histidine kinase CreC	-2.9
STM0651	STM0651	2-keto-3-deoxygluconate permease	-2.9
STM2030	*cbiT*	cobalt-precorrin-6Y C(15)-methyltransferase	-2.9
STM4473	*yjgM*	acetyltransferase	-2.9
STM1327	*ydiY*	outer membrane protein	-2.9
STM0657	*ybeU*	hypothetical protein	-2.9
STM4051	STM4051	outer membrane protein	-2.9
STM3693	*lldR*	DNA-binding transcriptional repressor LldR	-2.9
STM0707	*kdpF*	potassium-transporting ATPase subunit F	-2.9
STM1900	*ntpA*	dATP pyrophosphohydrolase	-2.9
STM0044	*yaaY*	hypothetical protein	-2.9
STM3444	*bfd*	bacterioferritin-associated ferredoxin	-2.9
STM1046	STM1046	tail assembly protein	-2.9
STM1040	STM1040	minor tail protein	-2.9
STM0780	STM0780	hypothetical protein	-2.9
STM2249	*ccmF*	cytochrome c-type biogenesis protein	-2.9
STM4382	*yjfR*	L-ascorbate 6-phosphate lactonase	-2.9

STM0035	STM0035	arylsulfatase	-2.9
STM2807	*nrdE*	ribonucleotide-diphosphate reductase subunit alpha	-2.9
STM1910	STM1910	penicillin-binding protein	-2.9
STM0704	*kdpC*	potassium-transporting ATPase subunit C	-2.9
STM1139	*csgG*	curli operon transcriptional regulator	-2.9
STM4196	STM4196	hypothetical protein	-2.9
STM4514.S	*yjiH*	inner membrane protein	-2.9
STM1032	STM1032	hypothetical protein	-2.9
STM2808	*nrdF*	ribonucleotide-diphosphate reductase subunit beta	-2.9
STM1031	STM1031	hypothetical protein	-2.8
STM1696	*sapF*	peptide transport protein	-2.8
STM0993	*mukE*	condesin subunit E	-2.7
STM3114	*speC*	ornithine decarboxylase	-2.7
STM3588	*yhiN*	hypothetical protein	-2.7
STM4076	*ydeZ*	sugar transport protein	-2.7
STM2499.S	*purM*	phosphoribosylaminoimidazole synthetase	-2.7
STM3872	*atpI*	F0F1 ATP synthase subunit I	-2.6
STM3242	*tdcD*	propionate/acetate kinase	-2.6
STM4538	STM4538	PTS permease	-2.6
STM1905	*yecO*	SAM-dependent methyltransferase	-2.6
STM0171	*yadF*	carbonic anhydrase	-2.6
STM3657	STM3657	outer membrane lipoprotein	-2.6
STM4266	*soxR*	redox-sensing transcriptional activator	-2.6
STM0419	*thiL*	thiamine monophosphate kinase	-2.6
STM1516	*ydeE*	MFS-type transporter YdeE	-2.6
STM3527	STM3527	hypothetical protein	-2.6
STM3971	*yigP*	inner membrane protein	-2.6
STM2434	STM2434	hypothetical protein	-2.6
STM3144	*hypA*	hydrogenase nickel incorporation protein HybF	-2.6
STM4481	*idnR*	L-idonate regulator	-2.6
STM1908	*yecM*	hypothetical protein	-2.6
STM1701	*yciW*	hypothetical protein	-2.6
STM2530	STM2530	anaerobic dimethylsulfoxide reductase	-2.6
STM1911	STM1911	hypothetical protein	-2.6
STM1083	*yccX*	acylphosphatase	-2.6
STM3628	*dppC*	dipeptide transporter	-2.6
STM3064	*iciA*	chromosome replication initiation inhibitor protein	-2.6
STM3623	*yhjT*	inner membrane protein	-2.6
STM1515	*ydeI*	hypothetical protein	-2.5
STM1392	*ssrA*	sensor kinase	-2.5
STM1754	*ychK*	hypothetical protein	-2.5
STM4286	*lpxO*	dioxygenase	-2.5
STM3585	*yhhJ*	ABC transport protein	-2.5
STM4550	*fhuF*	ferric hydroximate transport ferric iron reductase	-2.5
STM0111	*leuC*	isopropylmalate isomerase large subunit	-2.5

STM1496	STM1496	dimethylsulfoxide reductase	-2.5
STM1153	*msyB*	hypothetical protein	-2.5
STM0838	*ybiT*	ABC transporter ATPase	-2.5
STM0395	*sbcC*	exonuclease subunit SbcC	-2.5
STM0050	STM0050	nitrite reductase	-2.5
STM3868	*atpH*	F0F1 ATP synthase subunit delta	-2.5
STM0306	STM0306	adhesin/invasin protein PagN	-2.4
STM0363	STM0363	transcriptional regulator	-2.4
STM2498	*upp*	uracil phosphoribosyltransferase	-2.4
STM3555	*ugpE*	glycerol-3-phosphate transporter membrane protein	-2.4
STM2913	STM2913	permease	-2.4
STM0244	*rcsF*	outer membrane lipoprotein	-2.4
STM0431	*phnW*	2-aminoethylphosphonate--pyruvate transaminase	-2.4
STM1068	*lonH*	protease	-2.4
STM1868A	STM1868A	lytic enzyme	-2.4
STM0820	*rhlE*	ATP-dependent RNA helicase RhlE	-2.4
STM4574	STM4574	outer membrane protein	-2.4
STM0874	*mdaA*	nitroreductase A	-2.4
STM4316	STM4316	hypothetical protein	-2.4
STM2088	*rfbX*	O-antigen transferase	-2.4
STM1437	*ydhM*	transcriptional repressor	-2.4
STM0353	STM0353	cation transport ATPase	-2.4
STM1250	STM1250	hypothetical protein	-2.4
STM4066	STM4066	aminoimidazole riboside kinase	-2.4
STM1388	*orf70*	hypothetical protein	-2.4
STM4457	STM4457	transposase	-2.4
STM1767	*narL*	transcriptional regulator NarL	-2.4
STM2940	STM2940	hypothetical protein	-2.4
STM0510	*sfbA*	ABC transporter ATPase	-2.4
STM1598	*ydcR*	regulatory protein	-2.4
STM1593	*srfA*	virulence protein	-2.4
STM4347	*yjeP*	hypothetical protein	-2.4
STM3205	*uppP*	undecaprenyl pyrophosphate phosphatase	-2.4
STM4555	*leuQ*	tRNA-Leu	-2.4
STM0514	*ybbS*	DNA-binding transcriptional activator AllS	-2.4
STM1198	*pabC*	4-amino-4-deoxychorismate lyase	-2.4
STM0992	*mukF*	condesin subunit F	-2.4
STM2453	STM2453	hypothetical protein	-2.4
STM0159	STM0159	restriction endonuclease	-2.4
STM1193	*fabH*	3-oxoacyl-ACP synthase	-2.4
STM1082	STM1082	regulatory protein	-2.3
STM4588	*creB*	DNA-binding response regulator CreB	-2.3
STM2349	*yfcG*	glutathione S-transferase	-2.3
STM0145	*nadC*	quinolinate phosphoribosyltransferase	-2.3
STM0094	*djlA*	Dna-J like membrane chaperone protein	-2.3

STM0184	*pcnB*	poly(A) polymerase I	-2.3
STM2111	*wcaE*	glycosyl transferase family protein	-2.3
STM1823	*yoaH*	hypothetical protein	-2.3
STM2082	*rfbP*	undecaprenol-phosphate galactosephosphotransferase/O-antigen transferase	-2.3
STM1738	*yciI*	YciI-like protein	-2.3
STM3237	*yhaL*	hypothetical protein	-2.3
STM3315	*yrbH*	D-arabinose 5-phosphate isomerase	-2.3
STM0871	*ybjM*	inner membrane protein	-2.3
STM2930	*ispD*	2-C-methyl-D-erythritol 4-phosphate cytidylyltransferase	-2.3
STM0311	*yafJ*	glutamine amidotransferase	-2.3
STM4398	*cycA*	D-alanine/D-serine/glycine permease	-2.3
STM2958	*barA*	hybrid sensory histidine kinase BarA	-2.3
STM3129	STM3129	NAD-dependent aldehyde dehydrogenase	-2.3
STM2674	*trmD*	tRNA (guanine-N(1)-)-methyltransferase	-2.2
STM2480	*narQ*	nitrate/nitrite sensor protein NarQ	-2.2
STM3928	*wecF*	common antigen polymerase	-2.2
STM3164	*yqhD*	alcohol dehydrogenase	-2.2
STM3528	STM3528	phosphate-binding protein	-2.2
STM1984	*yodD*	hypothetical protein	-2.2
STM0938	*ybjE*	inner membrane protein	-2.2
STM3503	*greB*	transcription elongation factor GreB	-2.2
STM3524	*glpG*	intramembrane serine protease GlpG	-2.2
STM0986	*ycaQ*	hypothetical protein	-2.2
STM3944	STM3944	inner membrane protein	-2.2
STM0727	STM0727	hypothetical protein	-2.2
STM4056.S	*yiiM*	hypothetical protein	-2.2
STM3847	*yidY*	multidrug efflux system protein MdtL	-2.2
STM0847	*ybiK*	L-asparaginase	-2.2
STM1151	*mdoH*	glucosyltransferase MdoH	-2.2
STM1011	STM1011	hypothetical protein	-2.2
STM0678	*leuW*	tRNA-Leu	-2.2
STM0535	*lpxH*	UDP-2,3-diacylglucosamine hydrolase	-2.2
STM3607	*yhjC*	transcriptional regulator	-2.2
STM3549	STM3549	inner membrane protein	-2.2
STM3989	*ileT*	tRNA-Ile	-2.2
STM3560	*livF*	leucine/isoleucine/valine transporter ATP-binding subunit	-2.2
STM2312	*elaA*	hypothetical protein	-2.2
STM0584	*entD*	phosphopantetheinyltransferase component of enterobactin synthase multienzyme complex	-2.2
STM0936	*hcr*	HCP oxidoreductase	-2.2
STM4079.S	*yneC*	autoinducer-2 (AI-2) modifying protein LsrG	-2.2
STM3956	*yigI*	hypothetical protein	-2.2
STM2254	*ccmA*	cytochrome c biogenesis protein CcmA	-2.2
STM4420	STM4420	inner membrane protein	-2.2
STM2783	*nixA*	nickel transporter	-2.2

STM1517	*ydeD*	O-acetylserine/cysteine export protein	-2.2
STM2796	*yqaE*	transporter	-2.2
STM3215	*yqjI*	transcriptional regulator	-2.2
STM4376	*yjfC*	glutathionylspermidine synthase	-2.2
STM1613	STM1613	PTS system enzymeIIB component	-2.2
STM1618	STM1618	transcriptional repressor of sgc operon	-2.2
STM1322	*yniC*	2-deoxyglucose-6-phosphatase	-2.2
STM3113	*nupG*	nucleoside transport	-2.2
STM2439	*yfeL*	membrane carboxypeptidase	-2.2
STM0359	STM0359	hypothetical protein	-2.2
STM0345	STM0345	inner membrane protein	-2.2
STM0343	STM0343	hypothetical protein	-2.2
STM4517	*yjiO*	transporter	-2.2
STM3098	STM3098	transcriptional regulator	-2.2
STM4523	*yjiW*	endoribonuclease SymE	-2.2
STM2252	*ccmC*	heme exporter protein	-2.2
STM2128	*yegO*	multidrug efflux system subunit MdtC	-2.2
STM4477	*pepA*	leucyl aminopeptidase	-2.2
STM4353	*glyX*	tRNA-Gly	-2.2
STM4423	STM4423	DNA-binding protein	-2.2
STM4451	*nrdG*	anaerobic ribonucleotide reductase-activating protein	-2.2
STM2854	*hypA*	hydrogenase nickel incorporation protein	-2.2
STM1482	*ydgF*	multidrug efflux system protein MdtJ	-2.2
STM4472	*ytgA*	inner membrane protein	-2.2
STM1390	*orf242*	regulatory protein	-2.2
STM2294	*yfaZ*	inner membrane protein	-2.2
STM3324	*ptsO*	phosphohistidinoprotein-hexose phosphotransferase component of N-regulated PTS system (Npr)	-2.2
STM2008	STM2008	hypothetical protein	-2.2
STM3374.1n	STM3374.1n	hypothetical protein	-2.2
STM2262	*eco*	ecotin	-2.2
STM1472	STM1472	hypothetical protein	-2.2
STM1826	*sdaA*	L-serine deaminase I/L-threonine deaminase I	-2.1
STM1814	*minC*	septum formation inhibitor	-2.1
STM2944	*ygcB*	helicase	-2.1
STM1307	*astE*	succinylglutamate desuccinylase	-2.1
STM1049	STM1049	tail fiber protein	-2.1
STM3112	*mltC*	murein transglycosylase C	-2.1
STM3593	*yhiQ*	methyltransferase	-2.1
STM3334	STM3334	cytosine deaminase	-2.1
STM2364	*dedD*	hypothetical protein	-2.1
STM3498	*hslO*	Hsp33-like chaperonin	-2.1
STM4294	*yjdE*	arginine:agmatin antiporter	-2.1
STM1691	*pspF*	phage shock protein operon transcriptional activator	-2.1
STM2685	*smpA*	hypothetical protein	-2.1

STM3794	STM3794	regulatory protein	-2.1
STM1617	STM1617	epimerase	-2.1
STM3955	*rarD*	chloramphenicol resistance	-2.1
STM3619	*bcsA*	cellulose synthase catalytic subunit	-2.1
STM2858	*hypE*	hydrogenase formation protein	-2.1
STM2282	*glpQ*	glycerophosphodiester phosphodiesterase	-2.1
STM0212	STM0212	inner membrane protein	-2.1
STM2487	*purC*	phosphoribosylaminoimidazole-succinocarboxamide synthase	-2.1
STM0760	*aroG*	phospho-2-dehydro-3-deoxyheptonate aldolase	-2.1
STM1355	*ydiP*	transcriptional regulator	-2.0
STM0333	STM0333	transcriptional regulator	-2.0
STM3900	*ilvL*	ilvG operon leader peptide	-2.0
STM0801	*ybhK*	hypothetical protein	-2.0
STM3754	STM3754	hypothetical protein	-2.0
STM4223	*yjbF*	outer membrane lipoprotein	-2.0
STM0086	*kefC*	glutathione-regulated potassium-efflux system protein KefC	-2.0
STM2584	*gogB*	hypothetical protein	-2.0
STM2157	*yehS*	hypothetical protein	-2.0
STM1582	*nhoA*	arylamine N-acetyltransferase	-2.0
STM1931	*araH*	intracellular protease/amidase	-2.0
STM1797	*ymgE*	transglycosylase-associated protein	-2.0
STM3946	*yifL*	outer membrane lipoprotein	-2.0
STM0459	*ybaO*	transcriptional regulator	-2.0
STM0424	*xseB*	exodeoxyribonuclease VII small subunit	-2.0
STM1450	*pdxY*	pyridoxamine kinase	-2.0
STM2016	*cobT*	nicotinate-nucleotide--dimethylbenzimidazole phosphoribosyltransferase	-2.0
STM2485	*ypfI*	acetyltransferase	-2.0
STM3799	STM3799	hypothetical protein	-2.0
STM2596	STM2596	minor tail-like protein	-2.0
STM1489	*bioD*	dithiobiotin synthetase	-2.0
**Up-regulated genes**			
**Locus**	**Gene name**	**Function**	**Fold change**
STM3933	*leuT*	tRNA-Leu	12.3
STM1399	*sscA*	secretion system chaperone	12.3
STM3890	*gltU*	tRNA-Glu	10.5
STM3238	*yhaN*	inner membrane protein	9.6
STM3350	STM3350	inner membrane protein	8.9
STM3829	*dgoK*	2-oxo-3-deoxygalactonate kinase	8.2
STM1280	*yeaL*	inner membrane protein	8.2
STM0642	*ybeB*	hypothetical protein	6.8
STM4516	*yjiN*	inner membrane protein	6.8
STM2782	*mig-14*	transcriptional activator	6.8
STM0173	*yadH*	transporter	6.8
STM2842	*hypF*	hydrogenase maturation protein	6.8
STM0163	*pdxA*	4-hydroxythreonine-4-phosphate dehydrogenase	6.5

STM0135	*yacA*	SecA regulator SecM	6.2
STM2448	*yfeZ*	inner membrane protein	5.5
STM3932	*hisR*	tRNA-His	5.5
STM0387	*yaiI*	hypothetical protein	5.5
STM4549	STM4549	hypothetical protein	5.5
STM3133	STM3133	amidohydrolase	5.5
STM4593	*sthB*	fimbrial usher protein	5.5
STM0268	STM0268	hypothetical protein	5.5
STM1050	STM1050	tail fiber assembly like-protein	5.5
STM2288	STM2288	hypothetical protein	5.5
STM0183	*folK*	2-amino-4-hydroxy-6- hydroxymethyldihydropteridine pyrophosphokinase	5.5
STM0006	*yaaJ*	alanine/glycine transport protein	5.5
STM0001	*thrL*	thr operon leader peptide	4.8
STM1352	*ydiS*	hypothetical protein	4.8
STM1269	STM1269	chorismate mutase	4.8
STM3645	*yiaD*	outer membrane lipoprotein	4.8
STM2836	*gutM*	DNA-binding transcriptional activator GutM	4.8
STM4298	*melA*	alpha-galactosidase	4.8
STM0511	*sfbB*	ABC transporter ATPase	4.6
STM2628	STM2628	regulatory protein	4.5
STM0991	*smtA*	metallothionein SmtA	4.4
STM3169	STM3169	periplasmic dicarboxylate-binding protein	4.4
STM1762	*narJ*	nitrate reductase 1 delta subunit	4.4
STM0095	*rluA*	23S rRNA/tRNA pseudouridine synthase A	4.4
STM4571	STM4571	outer membrane protein	4.1
STM2139	STM2139	inner membrane protein	4.1
STM1874	STM1874	inner membrane protein	4.1
STM1877	STM1877	amidohydrolase	4.1
STM2612	STM2612	morphogenesis-like protein	4.1
STM2503	STM2503	diguanylate cyclase	4.1
STM2747	STM2747	hypothetical protein	4.1
STM3387	*yhdU*	hypothetical protein	4.1
STM0907	STM0907	chitinase	4.1
STM0263	*rnhA*	ribonuclease H	4.1
STM1523	*yneJ*	transcriptional regulator	4.1
STM4278.S	*nrfB*	cytochrome c nitrite reductase pentaheme subunit	4.1
STM0646	*holA*	DNA polymerase III subunit delta	4.1
STM0794	*bioB*	biotin synthetase	4.1
STM3275.S	*yhbV*	protease	4.1
STM1442	*ydhJ*	multidrug resistance efflux pump	4.1
STM0859	STM0859	transcriptional regulator	4.1
STM3792	STM3792	L-fucose permease	4.1
STM4399	*ytfE*	iron-sulfur cluster repair di-iron protein	4.1
STM1974	*fliK*	flagellar hook-length control protein	3.9
STM2306	*menC*	O-succinylbenzoate synthase	3.8

STM4469	*argI*	ornithine carbamoyltransferase subunit I	3.8
STM0821	*dinG*	ATP-dependent DNA helicase DinG	3.7
STM1397	*sseA*	secretion system chaperone protein	3.7
STM2202	*yeiH*	inner membrane protein	3.7
STM3986	*trkH*	potassium transporter	3.7
STM4287.S	*phnO*	aminoalkylphosphonic acid N-acetyltransferase	3.7
STM4591	*sthE*	major fimbrial subunit	3.4
STM4554	*leuP*	tRNA-Leu	3.4
STM0185	*yadB*	glutamyl-Q tRNA(Asp) synthetase	3.4
STM1973	*fliJ*	flagellar biosynthesis chaperone	3.4
STM2938	STM2938	hypothetical protein	3.4
STM4308	STM4308	anaerobic dehydrogenase component	3.4
STM4435	STM4435	hypothetical protein	3.4
STM1522	*ydeA*	sugar efflux transporter	3.4
STM1794	STM1794	membrane protein	3.4
STM4319	*phoN*	non-specific acid phosphatase	3.4
STM1549	STM1549	translation initiation inhibitor	3.4
STM3178	*ygiY*	sensor protein QseC	3.4
STM3168	*ygiR*	hypothetical protein	3.4
STM1821	*yoaA*	DNA helicase	3.4
STM1913	*flhA*	flagellar biosynthesis protein FlhA	3.4
STM0229	*lpxB*	lipid-A-disaccharide synthase	3.4
STM4511	*yjiE*	DNA-binding transcriptional regulator	3.4
STM3441	*rpsJ*	30S ribosomal protein S10	3.4
STM0023	*bcfC*	fimbrial usher	3.4
STM2394	*argW*	tRNA-Arg	3.4
STM4263	*yjcB*	inner membrane protein	3.4
STM4186	STM4186	hypothetical protein	3.4
STM3828	*dgoA*	galactonate dehydratase	3.4
STM2794	*ygaE*	DNA-binding transcriptional regulator CsiR	3.4
STM2496	*yfgE*	DNA replication initiation factor	3.4
STM1994	STM1994	inner membrane protein	3.4
STM0681	*nagD*	UMP phosphatase	3.3
STM0881	*ybjO*	inner membrane protein	3.2
STM0254	*aspU*	tRNA-Asp	3.2
STM2945	*sopD*	secreted effector protein	3.1
STM0877	*potF*	putrescine ABC transporter periplasmic-binding protein	3.1
STM1134	*serX*	tRNA-Ser	3.1
STM4090	*menA*	1,4-dihydroxy-2-naphthoate octaprenyltransferase	3.1
STM3346	*yhcM*	ATPase	3.1
STM1228	STM1228	hypothetical protein	3.1
STM3612	*kdgK*	ketodeoxygluconokinase	3.1
STM0041	STM0041	glycosyl hydrolase	3.1
STM2199	*cirA*	colicin I receptor	3.1
STM2654	*kgtP*	alpha-ketoglutarate transporter	3.1

STM1170	*mviN*	virulence protein	3.0
STM4508	*trpS2*	tryptophanyl-tRNA synthetase II	3.0
STM3118	STM3118	acetyl-CoA hydrolase	3.0
STM3926	*wzxE*	O-antigen translocase	3.0
STM3644	*bisC*	biotin sulfoxide reductase	3.0
STM1202	*ycfH*	metallodependent hydrolase	3.0
STM3475	*nirD*	nitrite reductase small subunit	3.0
STM2026	*cbiJ*	cobalt-precorrin-6x reductase	3.0
STM3765	*yicL*	permease	3.0
STM2570	STM2570	phosphotransferase system IIB component	3.0
STM4252	STM4252	inner membrane protein	3.0
STM2129	*yegB*	multidrug efflux system protein MdtE	2.9
STM1693	*sapB*	peptide transport protein	2.9
STM1491	*osmV*	proline/glycine betaine transport systems	2.9
STM1499	STM1499	dimethyl sulfoxide reductase subunit A	2.8
STM0201	STM0201	outer membrane protein	2.7
STM2309	*menD*	2-succinyl-5-enolpyruvyl-6-hydroxy-3- cyclohexene-1-carboxylate synthase	2.7
STM1687	*pspD*	peripheral inner membrane phage-shock protein	2.7
STM2021	*cbiQ*	vitamin B12 biosynthetic protein	2.7
STM1024	STM1024	hypothetical protein	2.7
STM1042	STM1042	minor tail protein	2.7
STM2679	*yfjD*	hypothetical protein	2.7
STM1084	*yccK*	sulfur transfer protein TusE	2.7
STM4031	STM4031	hypothetical protein	2.7
STM0626	*dpiA*	two-component response regulator DpiA	2.7
STM0621	*citF*	citrate lyase alpha chain/citrate-ACP transferase	2.7
STM1105	*hpaH*	4-hydroxyphenylacetate catabolism	2.7
STM0580	STM0580	regulatory protein	2.7
STM4112	*frwC*	fructose-like permease EIIC subunit 2	2.7
STM0564	STM0564	pyridine nucleotide-disulfide oxidoreductase	2.7
STM2143	*yegU*	glycohydrolase	2.7
STM1201	*holB*	DNA polymerase III subunit delta'	2.7
STM3574	*yhhM*	inner membrane protein	2.7
STM4230	*malK*	maltose/maltodextrin transporter ATP-binding protein	2.7
STM0396	*sbcD*	exonuclease subunit SbcD	2.7
STM3102	*yggU*	hypothetical protein	2.7
STM2223	*yejH*	ATP-dependent helicase	2.7
STM0303	*ybeJ*	xylanase/chitin deacetylase	2.7
STM3222	*ygjQ*	integral membrane protein	2.7
STM0331	STM0331	fumarylacetoacetate hydrolase	2.7
STM3284	*truB*	tRNA pseudouridine synthase B	2.7
STM2031	*cbiE*	cobalt-precorrin-6Y C(5)-methyltransferase	2.7
STM2887	*spaS*	surface presentation of antigens protein SpaS	2.7
STM1410	STM1410	hypothetical protein	2.7

STM1218	*lolD*	lipoprotein transporter ATP-binding subunit	2.7
STM3332	*yhcG*	hypothetical protein	2.7
STM2084	*rfbM*	mannose-1-phosphate guanylyltransferase	2.7
STM3949	*xerC*	site-specific tyrosine recombinase XerC	2.6
STM4032	STM4032	acetyl esterase	2.6
STM1709	*yciS*	inner membrane protein	2.6
STM1586	STM1586	hypothetical protein	2.6
STM3372	*mreD*	rod shape-determining protein MreD	2.6
STM3712	*rfaC*	ADP-heptose--LPS heptosyltransferase 1	2.5
STM2505	STM2505	inner membrane protein	2.5
STM4033	STM4033	regulatory protein	2.5
STM3608	*yhjD*	tRNA-processing ribonuclease	2.5
STM0426	*phnV*	2-aminoethylphosphonate transporter	2.5
STM4121	*argC*	N-acetyl-gamma-glutamyl-phosphate reductase	2.5
STM1379	*orf48*	amino acid permease	2.5
STM0968	*ycaD*	MFS family transporter protein	2.5
STM0846	*moeA*	molybdopterin biosynthesis protein MoeA	2.5
STM3681	STM3681	transcriptional regulator	2.5
STM2597	STM2597	major tail-like protein	2.4
STM1498	STM1498	dimethyl sulfoxide reductase	2.4
STM3952	*corA*	magnesium/nickel/cobalt transporter CorA	2.4
STM4228	*malF*	maltose transporter membrane protein	2.4
STM3764	*mgtC*	Mg2+ transport protein	2.4
STM2917	*ygbK*	tRNA synthase	2.4
STM2619	STM2619	hypothetical protein	2.4
STM0878	*potG*	putrescine ABC transporter ATP-binding protein	2.4
STM0382	STM0382	permease	2.4
STM3288	*yhbC*	hypothetical protein	2.4
STM2531	*pbpC*	penicillin-binding protein 1C	2.4
STM0410	STM0410	regulatory protein	2.4
STM1547	STM1547	transcriptional regulator	2.4
STM0802	*moaA*	molybdenum cofactor biosynthesis protein A	2.4
STM0406	*yajC*	preprotein translocase subunit YajC	2.4
STM3796A.S	STM3796A.S	integral membrane protein	2.4
STM0619	*citG*	triphosphoribosyl-dephospho-CoA synthase	2.4
STM4171	*yjaH*	inner membrane protein	2.4
STM3001	*thyA*	thymidylate synthase	2.4
STM2591	STM2591	tail assembly protein K-like	2.4
STM4107	*yijF*	hypothetical protein	2.4
STM1321	*ydjM*	hypothetical protein	2.4
STM3661	*xylA*	xylose isomerase	2.4
STM1706	*yciH*	translation initiation factor Sui1	2.4
STM1771	*chaA*	calcium/sodium:proton antiporter	2.4
STM3055	*gcvT*	glycine cleavage system aminomethyltransferase T	2.4
STM2768	STM2768	transposase	2.4

STM0618	*citT*	citrate/succinate transport antiport protein	2.4
STM1857	STM1857	acetyltransferase	2.4
STM2339	*yfcC*	hypothetical protein	2.4
STM2353	*hisQ*	histidine/lysine/arginine/ornithine transport protein	2.4
STM3241	*tdcE*	pyruvate formate-lyase 4/2-ketobutyrate formate-lyase	2.3
STM3523	*glpR*	DNA-binding transcriptional repressor GlpR	2.3
STM4229	*malE*	maltose ABC transporter periplasmic protein	2.3
STM2607	STM2607	head-to-tail joining-like protein	2.3
STM3199	*yqiK*	hypothetical protein	2.3
STM4485	*idnK*	D-gluconate kinase	2.3
STM4129	*trmA*	tRNA (uracil-5-)-methyltransferase	2.3
STM3089	*yqgD*	inner membrane protein	2.3
STM1893	*znuB*	high-affinity zinc transporter membrane protein	2.3
STM1806	*nhaB*	sodium/proton antiporter	2.3
STM2630	STM2630	hypothetical protein	2.3
STM4542	*yjjA*	hypothetical protein	2.3
STM0221	*uppS*	undecaprenyl pyrophosphate synthase	2.2
STM0950	STM0950	SlsA	2.2
STM1739	*cls*	cardiolipin synthetase	2.2
STM4317	STM4317	hypothetical protein	2.2
STM1605	*ydcN*	repressor	2.2
STM3373	*mreC*	rod shape-determining protein MreC	2.2
STM3467	*yhfK*	inner membrane protein	2.2
STM0081	STM0081	hypothetical protein	2.2
STM3341	*sspB*	ClpXP protease specificity-enhancing factor	2.2
STM3870	*atpE*	F0F1 ATP synthase subunit C	2.2
STM0260	*mltD*	membrane-bound lytic murein transglycosylase D	2.2
STM1937	*tyrP*	tyrosine-specific transport protein	2.2
STM0116	*ilvI*	acetolactate synthase 3 catalytic subunit	2.2
STM2506	STM2506	inner membrane protein	2.2
STM1136	*ycdX*	hydrolase	2.2
STM2663	*yfiO*	outer membrane protein assembly complex subunit YfiO	2.2
STM1059	*ycbW*	hypothetical protein	2.2
STM1731	STM1731	catalase	2.2
STM1735	*yciB*	intracellular septation protein A	2.2
STM1188	STM1188	inner membrane lipoprotein	2.1
STM4262	STM4262	ABC-type bacteriocin/lantibiotic exporter	2.1
STM4346	*yjeO*	inner membrane protein	2.1
STM1820	*yeaZ*	molecular chaperone	2.1
STM0285	STM0285	inner membrane protein	2.1
STM0287	STM0287	hypothetical protein	2.1
STM2275	STM2275	regulatory protein	2.1
STM3124	STM3124	response regulator	2.1
STM3126	STM3126	amino acid transporter	2.1
STM1835	*rrmA*	23S rRNA methyltransferase A	2.1

STM4320	STM4320	regulatory protein	2.1
STM1761	*narI*	nitrate reductase 1 gamma subunit	2.1
STM1773	*ychA*	transcriptional regulator	2.1
STM4365	*yjeT*	inner membrane protein	2.1
STM1898	*ruvC*	Holliday junction resolvase	2.1
STM2973	*fucO*	L-1,2-propanediol oxidoreductase	2.1
STM4335	*ecnA*	entericidin A precursor	2.1
STM2989	*metZ*	tRNA-Met	2.1
STM3079.S	STM3079.S	hydrolase/acyltransferase	2.1
STM4304	*dcuS*	sensory histidine kinase DcuS	2.1
STM2301	*arnT*	4-amino-4-deoxy-L-arabinose transferase	2.1
STM1789	STM1789	hydrogenase 1 maturation protease	2.1
STM3037	*glyU*	tRNA-Gly	2.1
STM1733	STM1733	ferredoxin	2.1
STM4143	*tyrU*	tRNA-Tyr	2.1
STM1401	*sseD*	translocation machinery component	2.1
STM3357	STM3357	regulatory protein	2.1
STM0481	*priC*	primosomal replication protein N"	2.1
STM4069	STM4069	hypothetical protein	2.1
STM0674	*glnV*	tRNA-Gln	2.1
STM3374	*mreB*	rod shape-determining protein MreB	2.1
STM0503	*ybbM*	transporter	2.1
STM3994	*mobA*	molybdopterin-guanine dinucleotide biosynthesis protein MobA	2.1
STM3667	*yiaJ*	transcriptional repressor	2.1
STM3714	*rfaK*	hexose transferase	2.1
STM0177	*stiA*	fimbrial subunit	2.1
STM0568	*pheP*	phenylalanine transporter	2.1
STM1089	STM1089	inner membrane protein	2.1
STM1093	STM1093	hypothetical protein	2.1
STM0581	STM0581	regulatory protein	2.1
STM2586	STM2586	phage tail assembly-like protein	2.1
STM0598	*entA*	2,3-dihydroxybenzoate-2,3-dehydrogenase	2.1
STM3531	STM3531	dihydroxyacid dehydratase	2.1
STM3182	*yqiA*	esterase YqiA	2.1
STM3451	*yheN*	sulfur transfer complex subunit TusD	2.1
STM4178	*gltV*	tRNA-Glu	2.1
STM4449	STM4449	bifunctional antitoxin/transcriptional repressor RelB	2.1
STM0866	*mdfA*	multidrug translocase	2.1
STM0835	STM0835	manganese transport regulator MntR	2.1
STM4594	*sthA*	fimbrial chaperone	2.1
STM3821	*torD*	chaperone protein TorD	2.1
STM3805	*yidH*	inner membrane protein	2.1
STM2824	STM2824	tRNA-Arg	2.1
STM2511	*guaB*	inosine 5'-monophosphate dehydrogenase	2.1
STM2099	*wcaM*	colanic acid biosynthesis protein	2.1

STM4247	*alr*	alanine racemase	2.1
STM2122	*udk*	uridine kinase	2.1
STM2125	*yegD*	chaperone	2.1
STM1488	*mlc*	pts operon transcriptional repressor	2.1
STM3732	*slmA*	nucleoid occlusion protein	2.1
STM3461	STM3461	hypothetical protein	2.0
STM2097	*rfbB*	dTDP-glucose-4,6-dehydratase	2.0
STM2145	*yegW*	regulatory protein	2.0
